# Transcription Factor Nrf1 Is Topologically Repartitioned across Membranes to Enable Target Gene Transactivation through Its Acidic Glucose-Responsive Domains

**DOI:** 10.1371/journal.pone.0093458

**Published:** 2014-04-02

**Authors:** Yiguo Zhang, Yonggang Ren, Shaojun Li, John D. Hayes

**Affiliations:** 1 The NSFC-funded Laboratory of Cell Biochemistry and Gene Regulation, College of Medical Bioengineering and Faculty of Life Sciences, Chongqing University, Chongqing, China; 2 Division of Cancer Research, Medical Research Institute, Ninewells Hospital & Medical School, University of Dundee, Scotland, United Kingdom; University of Saarland Medical School, Germany

## Abstract

The membrane-bound Nrf1 transcription factor regulates critical homeostatic and developmental genes. The conserved N-terminal homology box 1 (NHB1) sequence in Nrf1 targets the cap‘n’collar (CNC) basic basic-region leucine zipper (bZIP) factor to the endoplasmic reticulum (ER), but it is unknown how its activity is controlled topologically within membranes. Herein, we report a hitherto unknown mechanism by which the transactivation activity of Nrf1 is controlled through its membrane-topology. Thus after Nrf1 is anchored within ER membranes, its acidic transactivation domains (TADs), including the Asn/Ser/Thr-rich (NST) glycodomain situated between acidic domain 1 (AD1) and AD2, are transiently translocated into the lumen of the ER, where NST is glycosylated in the presence of glucose to yield an inactive 120-kDa Nrf1 glycoprotein. Subsequently, portions of the TADs partially repartition across membranes into the cyto/nucleoplasmic compartments, whereupon an active 95-kDa form of Nrf1 accumulates, a process that is more obvious in glucose-deprived cells and may involve deglycosylation. The repartitioning of Nrf1 out of membranes is monitored within this protein by its acidic-hydrophobic amphipathic glucose-responsive domains, particularly the Neh5L subdomain within AD1. Therefore, the membrane-topological organization of Nrf1 dictates its post-translational modifications (i.e. glycosylation, the putative deglycosylation and selective proteolysis), which together control its ability to transactivate target genes.

## Introduction

Defining the molecular details of membrane protein biogenesis is essential if we are to understand the functions of integral membrane proteins in the cell. By comparison with water-soluble proteins, membrane-protein topology defines both the biogenesis of fully-folded integral membrane proteins and their biological activity [1]. It is however unclear to what extent the normal functioning of membrane proteins is controlled topologically by their dynamic reorientation across membranes. The membrane-topogenic control of transmembrane transcription factors is complex, as they require to be released from the endoplasmic reticulum (ER) before they can translocate to the nucleus and interact with their target genes. The two prototypic membrane-bound transcription factors ATF6 and SREBP1 are trafficked from the ER into the Golgi apparatus, whereupon both are proteolytically processed through regulated intramembrane proteolysis (RIP) allowing them to be consecutively cleaved by Site-1 and Site-2 proteases [2], in order to allow their active N-terminal portions to be released from membranes prior to nuclear translocation [3], [4]. However, other membrane-bound transcription factors, such as certain cap‘n’collar (CNC)-basic basic-region leucine zipper (bZIP) family members [5]–[7], are not processed *via* RIP and it is unclear how their activity is regulated. Herein, we describe how the topology of a membrane-bound CNC transcription factor controls its activity.

**Figure 1:**
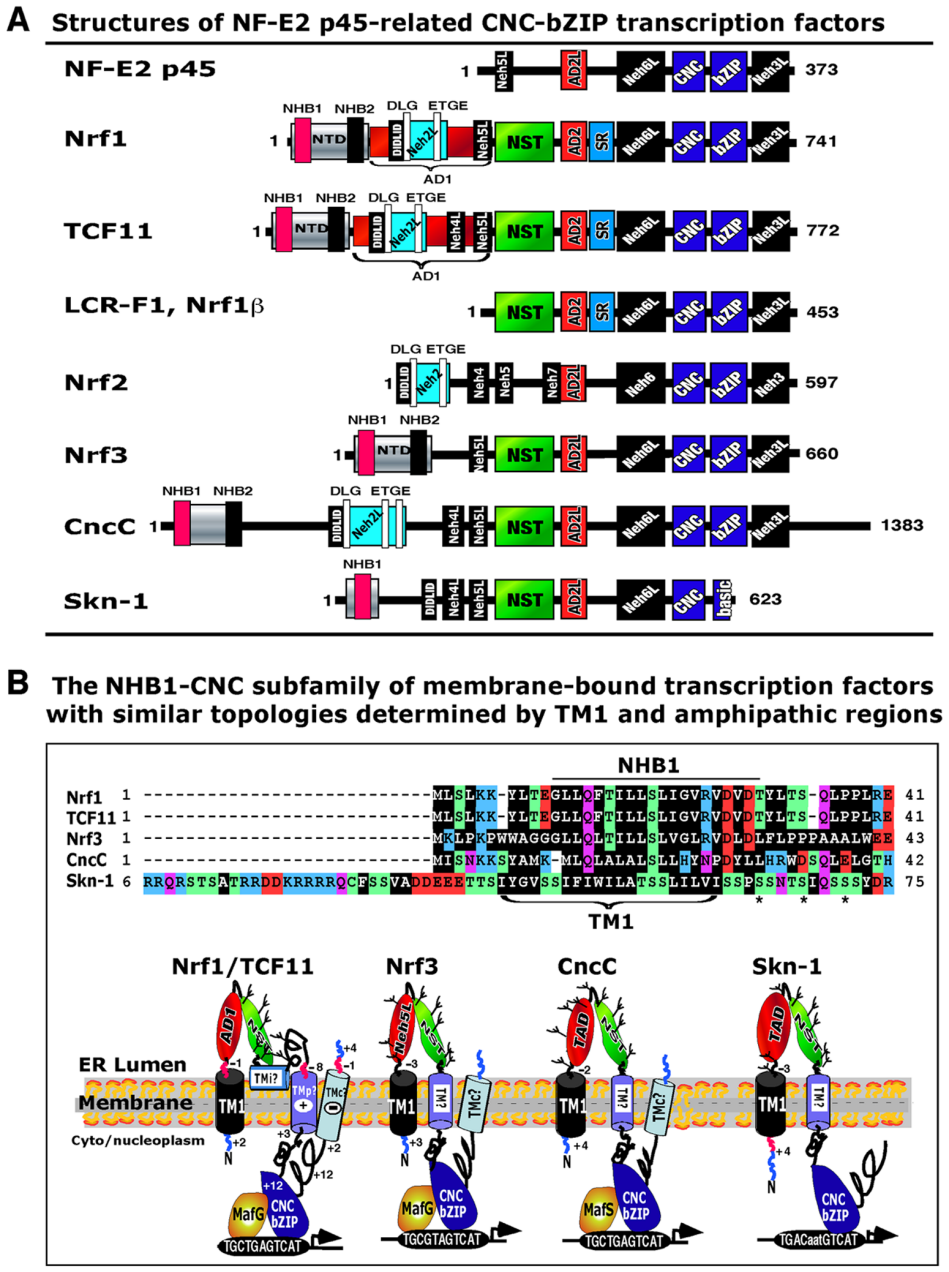
The NHB1-CNC subfamily of membrane-bound transcription factors. (**A**) The structural domains of NF-E2 p45-related CNC-bZIP transcription factors have been identified by bioinformatic analyses of their amino acid sequences. The Neh4 and Neh5 domains, which act as transactivation domains (TADs) in Nrf2 [49], [75], are represented by Neh4L and Neh5L in other family proteins. In Nrf1, AD1 is an essential TAD, containing the PEST1, Neh2L, CPD and Neh5L subdomains (see Text). Neh2L contains the DIDLID/DLG element and the ETGE motif; both are present in CncC and Nrf2 where they regulate protein stability. In addition to AD1, the AD2 region also functions as a TAD in Nrf1 [6] and is conserved amongst all other CNC family members, where it has been labeled AD2L. The ER-targeting NHB1 peptide of Nrf1/TCF11 and its NST glycodomain [7] are represented in Nrf3, CncC and Skn-1. We propose that Nrf1, Nrf3, CncC and Skn-1 constitute a subfamily of CNC transcription factors, called NHB1-CNC, which are membrane-bound proteins that are glycosylated in the lumen of the ER. For definition of the major acronyms, see Box S1. (**B**) The conserved topological structure of NHB1-CNC factors within and around membranes is predicted by bioinfomatics. Their ER-targeting mechanism has been confirmed in Nrf1, Nrf3 and CncC [5], [11], to occur *via* the conserved TM1 motif. The ability of NHB1-CNC factors (except Skn-1) to bind ARE sequences in target gene promoter regions is mediated through their CNC/bZIP domains that are retained on the cyto/nucleoplasmic side of membranes. The DNA-binding activity of Skn-1 is attributed to its CNC domain [76]. The TADs of the membrane-bound factors are transiently translocated into the luminal side of the ER during the initial co-translactional topogenesis. When these factors are required to activate their target genes, the luminal TADs are repartitioned and dislocated/retrotranslocated out of the luminal side across membranes into the cytoplasmic and/or nucleoplasmic compartments, where they are presented to the general transcriptional machinery before transactivating target gene expression. In addition, the asterisk* indicates the presence of putative GSK-3 phosphorylation sites in Nrf1, TCF11 and Skn-1.

The CNC-bZIP family of transcription factors controls homeostatic and developmental pathways by regulating the expression of genes encoding antioxidant proteins, detoxification enzymes, metabolic enzymes, and 26S proteosomal subunits [8]–[12]. This family comprises the *Drosophila* Cnc protein, the *Caenorhabditis elegans* Skn-1 protein, the vertebrate activator NF-E2 p45 and its related factors Nrf1 (including the long form TCF11 and the short isoform LCR-F1), Nrf2, and Nrf3 (Fig. 1A), and the repressors Bach1 and Bach2. In all cases except Skn-1, the CNC proteins heterodimerize with a small Maf or other bZIP proteins before they can bind to antioxidant/electrophile response element (ARE/EpRE) sequences in their target gene promoters [13]–[15].

Since the CNC family transcription factors were cloned from different metazoan species in the mid-1990s, research on them has grown rapidly [8]. However, there has been a disproportionate focus on the water-soluble Nrf2 factor and substantially less is known about the function of the membrane-bound Nrf1 factor. The lack of attention afforded Nrf1 is surprising given the fact that its global knockout in the mouse leads to embryonic lethality and severe oxidative stress [16]–[18]. Moreover, conditional knockout of Nrf1 in the liver, brain and bone results in non-alcoholic steatohepatitis and hepatoma [19], [20], neurodegeneration [21], [22], and reduced bone size [23], respectively. Surprisingly, Nrf2 is similarly considered to be a master regulator of adaptive responses to oxidative stressors and electrophiles [24], [25], but it is dispensable for development because global knockout of its gene in mice yields viable animals [26]. The fact that Nrf1, but not Nrf2, is essential for maintaining cellular homeostasis and organ integrity, indicates that it fulfils a unique and indispensable function(s).

Amongst CNC-bZIP proteins, an N-terminal homology box 1 (NHB1)-CNC subfamily exists that comprises membrane-bound transcription factors (Fig. 1B). These include Nrf1/TCF11 [6], [7], [9], Nrf3 [5], CncC [11] and Skn-1 [27], all of which possess an NHB1 signal peptide that targets them to the ER and provides a similar topology within and around membranes. Most of the NHB1-CNC factors lack a signal peptidase cleavage site and therefore cannot be released from the ER [5], [7], suggesting the existence of a novel mechanism(s) that regulates their activity. As an example, Nrf1 is anchored within the ER membrane through the TM1 region (aa 7-26) within its NTD, and is either retained therein or sorted out into the nuclear envelope membranes, where it gains access to target genes in order to mediate transcriptional responses to redox stress or glucose deprivation [6], [28]. The overall membrane-topology of Nrf1 is determined by TM1 [7], [29] in cooperation with other semihydrophobic amphipathic regions (Fig. S1), but it is evidently distinct from those of ATF6 and SREBP1. It is notable that Nrf1 is also subject to ER-associated degradation (ERAD) and that involves retrotranslocation from the ER to the cytoplasm [30], but this does not result in transactivation of ARE-driven gene expression.

In the present study we have discovered a hitherto unknown mechanism by which the membrane-topology of Nrf1 controls its transcriptional activity. When required, the acidic-hydrophobic amphipathic glucose-responsive TADs of Nrf1 are partially repartitioned from the luminal side of the ER across membranes, so that its NST-flanked AD1 and AD2 are retrotranslocated into the cyto/nucleoplasmic compartments. As a consequence, Nrf1 is able to transactivate its target genes in the nucleus. In addition, we have investigated that the post-translational processing of Nrf1 affects the expression of ARE/EpRE-driven genes.

## Materials and Methods

### Chemicals and antibodies

All chemicals were of the highest quality commercially available, with the exception of the chitobiose-based PNGase inhibitors that were provided by Dr. Martin D Witte (Leiden University) [31]. The ER extraction kit was purchased from Sigma-Aldrich. PNGase F, Endo H and PK were obtained from New England Biolabs. Rabbit polyclonal antibodies against calreticulin (CRT) and green fluorescent protein (GFP) were bought from Calbiochem and Abcam PLC, respectively. A mouse monoclonal antibody against the V5 epitope and rabbit polyclonal antibodies against DsRed (a *Discosoma sp*. red fluorescent protein) were from Invitrogen Ltd. Antisera against Nrf1were produced in rabbits using a polypeptide covering aa 292-741.

### Expression constructs

Expression constructs for full-length mouse Nrf1 have been described previously [6], [28]. Mutants were created by PCR-directed point or deletion mutagenesis within the TADs, SR/PEST2 or Neh6L of Nrf1, as described previously [32]. The sandwich fusion protein DsRed/N275/GFP was engineered by inserting the cDNA sequence encoding the N-terminal 275 amino acids of Nrf1 (N275) between the cDNAs for DsRed2 and GFP within the pDsRed2-GFP vector through the SalI/KpnI site [6]. The fidelity of all cDNA products was confirmed by sequencing.

### Cell culture, transfection, and reporter gene assays

Equal numbers (3×10^5^) of monkey kidney COS-1 cells (purchased from ATCC and maintained in our laboratory) were seeded in 6-well plates and grown for 24 h in DMEM containing 25 mM glucose and 10% FBS. After reaching 70% confluence, the cells were transfected with a Lipofectamine 2000 (Invitrogen) mixture that contained an expression construct for wild-type Nrf1 or a mutant protein, together with *P_SV40_GSTA2*-6×ARE-Luc, which contains six copies of the core ARE consensus sequence from rat *GSTA2* [6], [28], [33], along with pcDNA4 HisMax/*lacZ* encoding β-galactosidase (β-gal) that was used as a control for transfection efficiency. In addition, mutant versions of these reporter genes that lacked the ARE sequence were used as negative controls. Luciferase or chloramphenicol acetyltransferase (CAT) activity was measured approximately 36 h after transfection. The basal and stimulated ARE-driven reporter gene activity obtained following transfection with an expression vector for Nrf1 (or its mutants) was calculated as a ratio of its value against the background activity (i.e. the luciferase acitivity obtained following co-transfection of an empty pcDNA3.1/V5 His B vector and an ARE-driven reporter after subtraction of the non-specific value from cotransfecting an empty pcDNA3.1/V5 His B vector and a non-ARE-containing Luc plasmid). Subsequently, the basal activity of full-length wild-type Nrf1 was given the value of 1.0, and other data were calculated as fold change (mean ± S.D) relative to this value. The data presented each represent at least 3 independent experiments undertaken on separate occasions that were each performed in triplicate. Differences in their transcriptional activity were subjected to statistical analyses.

### Subcellular fractionation followed by *in vitro* membrane protease protection assays as combined in dFPP

To investigate the topological folding of Nrf1 within membranes and its repartitioning out of membranes, intact ER-rich membrane and nuclear fractions were prepared from COS-1 cells expressing wild-type Nrf1, its fusion proteins or its mutants, and these were subjected to membrane PK protection assays, as described previously [34]–[38]. The intact ER-rich fraction was resuspended in 100 μl of 1× isotonic buffer. Subsequently, membrane proteinase protection reactions were performed for 15, 30 or 60 min on ice in an aliquot (50 μg of protein) of the membrane-containing preparation with proteinase K (PK) added to a final concentration of 50 or 100 μg protein/ml in either the presence or absence of 1% (v/v) Triton X-100 (TX). The reactions were stopped by incubation at 90°C for 10 min following the addition of 1 mM PMSF. Subsequently, the reaction products were analysed by western blotting with antibodies against either Nrf1β or the V5 ectope. The amount of Nrf1 protein remaining after PK digestion was calculated as described below. Importantly, fluorescence protease protection (FPP) assay [39] was performed to determine the topology of membrane proteins. Double fluorescence sandwiched protein linked to proteinase protection assays has been called dFPP herein.

### Live-cell imaging combined with *in vivo* membrane protease protection assays

COS-1 cells (10^6^) were seeded in 35-mm dishes and cultured overnight in 25 mM-glucose medium. The cells were then cotransfected for 6 h with 3 mg DNA of each expression construct for Nrf1/GFP [29] and 0.5 mg DNA encoding ER/DsRed, a luminal-resident protein marker of the ER [2], [28]. Subsequently, cells were allowed to recover from transfection for 16 h, and were then subjected to *in vivo* membrane protease protection assays, along with live cell imaging to determine dynamic movement of the luminal-resident protein from the ER into extra-lumininal compartments, whereupon the protein was not protected by membranes and thus was proteolytically digested by PK as reported elsewhere [36], [40]. Briefly, the plasma membranes of COS-1 cells were permeabilized by digitonin (20 mg/ml) for 10 min. Thereafter, the cells were subjected to *in vivo* membrane protection reactions against digestion by PK (50 mg/ml) for 35 min before addition of 0.1% (v/v) TX. During the experiment, live-cell images were acquired every min under a 40× objective lens mounted on Leica DMI 6000 green and red fluorescence microscopes equipped with a high-sensitivity Hamamatsh ORCA-ER camera, cell environment control units (at 37°C in 5% CO_2_ culture conditions) and a definitive focus module. Relative fluorescence units were measured using Simulator SP5 Multi-Detection system for GFP with 488-nm excitation and 507-nm emission, and for DsRed with 570-nm excitation and 650-nm emission.

### Gycosylation mapping, deglycosylation reactions and western blotting

The construct encoding Nrf1^(1-7)xN/Q^ cannot be glycosylated in the ER lumen [6]. Using the cDNA for Nrf1^(1-7)xN/Q^ as a template, a series of N-linked glycosylation asparagine acceptor sites were introduced into its AD1, AD2 and PEST2 regions. It was anticipated that, if the engineered glycosylation sites were translocated into the ER lumen, the mutant Nrf1 protein would be glycosylated by *in vivo* addition of a glycan precursor Glc3Man9GlcNAc2; this technique is called glycosylation mapping mutagenesis [41]. Modification of the engineered protein was detected by *in vitro* deglycosylation using 500 units of Endo H or PNGase F, followed by western blotting. On some occasions, nitrocellulose membranes that had already been blotted with an antibody were washed for 30 min with stripping buffer before being re-probed with an additional primary antibody against CRT or β-Actin; both served as internal controls to verify equal loading of protein into each electrophoretic well [42]. The intensity of blots was calculated using Quantity One software developed at Bio-Rad Laboratories.

### Bioinformatic analysis

The membrane-topology of Nrf1 was predicted using several bioinformatic algorithms, including the TopPred (http://mobyle. pasteur.fr/cgi-bin/portal.py?form = toppred), HeliQuest (http://heliquest.ipmc.cnrs.fr/) and AmphipaSeek (http://npsa-pbil.ibcp.fr/cgi-bin/npsa_automat.pl?page=/NPSA/npsa_amphipaseek.html) programmes. The PEST sequence, as a potential proteolytic cleavage site for proteasome and/or calpain, was found in Nrf1 using the ePESTfind program at http://mobyle.pasteur.fr/cgi-bin/portal.py?#forms::epestfind. The T-Coffee program was employed to align Nrf1 amino acid sequences with those of its orthologues or other known membrane-bound proteins.

### Statistical analysis

The statistical significance of changes in Nrf1 activity and the intensity of immunoblots was determined using the Student's *t* test or *M*ultiple *An*alysis *o*f *Va*riations (MANOVA). The data presented herein are shown as a fold change (mean ± S.D), each of which represents at least 3 independent experiments undertaken on separate occasions that were each performed in triplicate.

## Results

### The NST domain of Nrf1 is glycosylated in the ER lumen and is apparently deglycosylated after being repartitioned across membranes into cyto/nucleoplasmic compartments

N-linked glycosylation of proteins is catalyzed by oligosaccharyltransferases in the ER lumen [43], [44], whilst subsequent deglycosylation of N-linked glycoprotein occurs through enzymatic reactions catalyzed by peptide:N-glycosidases (PNGase) and/or endoglycosidases (Endo) in the extra-luminal cytoplasmic and/or nucleoplasmic subcellular compartments [41], [45]. In addition to removing N-linked glycans, PNGases also cleave glycan-attached amide group from each of the glycosylated Asn residues, to yield acidic Asp residues. Our previous work revealed that Nrf1 is glycosylated in the ER through its NST domain [7], and that this glycodomain, together with AD1 and AD2, contributes to transactivation activity [6]. We therefore postulate that the NST domain of Nrf1 functions as a *bona fide* TAD only after it has been repartitioned and retrotranslocated from the ER lumen, where it is glycosylated, into the cyto/nucleoplasm, where it is deglycosylated. In this scenario, deglycosylated Nrf1 would be a more acidic protein than its original non-glycosylated form because Asn (with its side chain pKa  =  0.0) residues are changed to Asp (pKa  =  3.9) residues [41], and might therefore be expected to increase transactivation activity because acidic residues responsible for a small 9-aa TAD element [46], [47] enable an interaction between acidic activators and the general transcriptional machinery (e.g. TBP-TF11A) [48].

**Figure 2:**
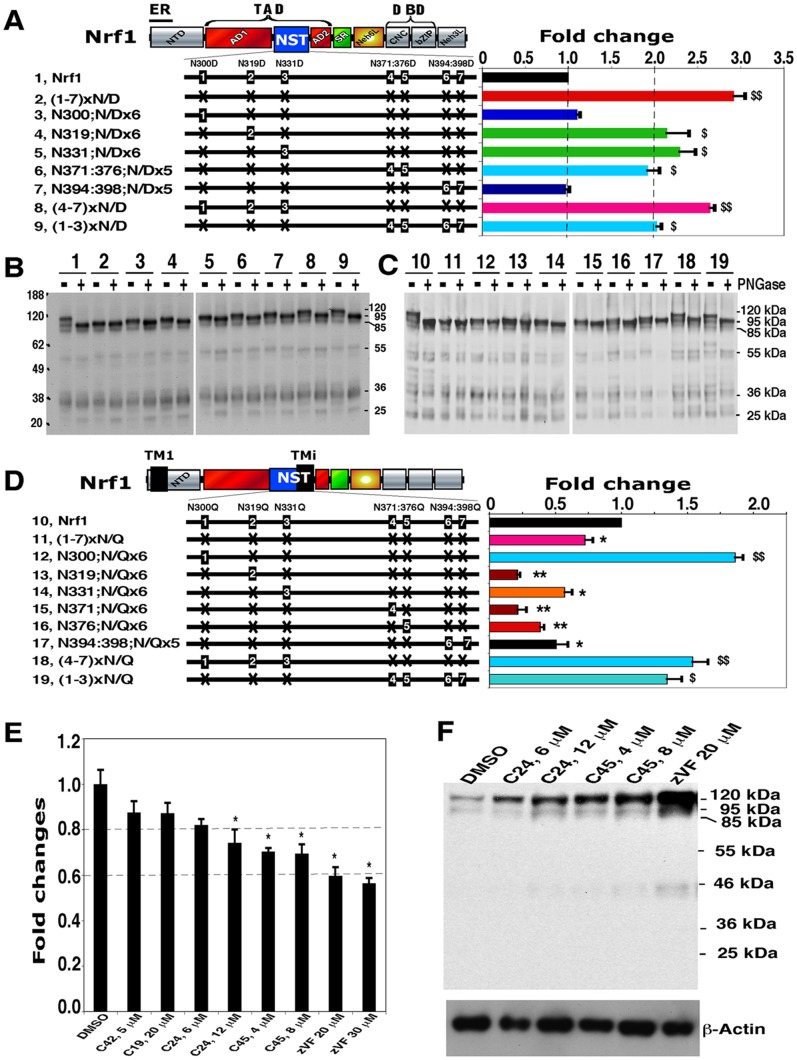
Regulation of Nrf1 by glycosylation and deglycosylation of its NST domain. (**A**) The *left* schematic illustrates structural domains of Nrf1 and its N/D-scanning mutants in the NST glycodomain. The *right* panel shows reporter gene activity measured after COS-1 cells had been cotransfected with each of expression constructs (1.2 μg), together with *P_SV40_GSTA2-*6×ARE-Luc (0.6 μg) and β-gal plasmid (0.2 μg), and allowed to recover in fresh media for an additional 24 h before lysis. The data were calculated as a fold change (mean ± S.D) of transactivation by N/D mutants of Nrf1, as described elsewhere [34]. Significant increases in activity, relative to wild-type Nrf1, are indicated: $, p<0.05 and $$, p<0.001, n = 9). (**B** and **C**) PNGase F-catalyzed deglycosylation was performed on total lysates of cells that expressed wild-type Nrf1, its N/D mutants (***B***, *lanes 2 to 9*) or N/Q mutants (***C***, *lanes 11 to 19*). The digest products were resolved by 4–12% LDS/NuPAGE and visualized by western blotting with V5 antibodies. (**D**) The *left* schematic depicts the N/Q-scanning mutants, and locations of the TM1 and TMi sequences (). The *right* panel shows the reporter gene activities produced by Nrf1 and its N/Q mutants. Significant decreases in activity are indicated: *, p<0.05 and **, p<0.001 (n = 9). (**E** and **F**) Inhibition of Nrf1 deglycosylation by C19, C24, C45 and Z-VAD-FMK (zVF) causes significant increases in the amount of the 120-kDa Nrf1 glycoprotein. COS-1 cells were cotransfected with an expression construct for wild-type Nrf1 or an empty vector (as a control), along with *P_SV40_GSTA2-*6×ARE-Luc and the β-gal plasmid. The cells were allowed to recover in fresh medium containing 5.5 mM glucose and 10% FBS for 8 h, before being treated for 18 h with the above chemicals in fresh medium with 10% dialyzed FBS that contained no added-glucose (i.e. ‘no-glucose’). Repression of Nrf1 activity by the PNGase inhibitors was analyzed by luciferase reporter assay (**E**), showing a significant difference (*p<0.05; n = 9) between the indicated inhibitors and DMSO. Expression of Nrf1 proteins was visualized by immunoblotting with V5 antibodies (**F**). β-actin was employed as an internal control for protein loading.

To test this hypothesis, asparagine-to-aspartate (N/D) mutagenesis was employed to determine whether reporter gene transactivation by Nrf1 is increased when the NST domain is more acidic (Fig. 2A, *left*). Within this domain, all seven glycosylation consensus sites were mutated to create Nrf1^(1-7)xN/D^. As anticipated, Nrf1^(1-7)xN/D^ exhibited almost 2.9-fold greater transactivation activity than the wild-type factor (Fig. 2A, *right columns 2 vs 1*). Western blotting of Nrf1^(1-7)xN/D^ showed that it possessed a faster electrophoretic mobility in NuPAGE gels than the wild-type protein, but the mobility of Nrf1^(1-7)xN/D^ was unchanged by prior incubation with PNGase F (Fig. 2B, *lanes 2 vs 1*). We therefore refer to Nrf1^(1-7)xN/D^ as the putative deglycosylated activated protein form. By contrast, other N/D-mutant proteins that retained between one and four of their Asn-glycosylation sites showed slower electrophoretic mobilities than Nrf1^(1-7)xN/D^ (*lanes 3* to *9 vs 2*). Following PNGase-catalyzed deglycosylation reactions, those N/D mutants that retained native Asn-glycosylation sites all exhibited faster electrophoretic mobilities, which resembled that of Nrf1^(1-7)xN/D^ (though the latter was slightly slower than the wild-type 95-kDa deglycosylated Nrf1). The increase in the electrophoretic mobility following deglycosylation revealed that all N/D-mutants except Nrf1^(1-7)xN/D^ were subject to varying levels of Asn-glycosylation, and they are thus assumed to have been translocated into the ER lumen where their NST domains were glycosylated.

Further examination of N/D mutants demonstrated that the enhanced transactivation activity of Nrf1^(1-7)xN/D^ was diminished substantially by glycosylation at N^300^ (in Nrf1^N300;N/Dx6^) or N^394^/N^398^ (in Nrf1^N394:398;N/Dx5^), though these two mutants still activated ARE-driven reporter activity to a similar extent as wild-type Nrf1. The high activity of Nrf1^(1-7)xN/D^ indicates it can increase gene transcription despite lacking glycosylation sites in its NST domain. Glycosylation of Nrf1 at N^300^ or N^394^/N^398^ (Fig. 2B, *lanes 3, 7*) was associated with a marked reduction in transactivation activity; N^300^ is located C-terminally to the Neh5L subdomain of AD1 (aa 280-298, that acts as an essential TAD [6], [49]), whereas N^394^ and N^398^ are situated C-terminally to the TMi peptide (aa 374-393) within the NST domain and N-terminally to the acidic hydrophobic region (aa 403-440) of AD2. These findings suggest that glycosylation of N^300^ and N^394^/N^398^ may modulate the positioning of the Neh5L subdomain, the TMi peptide and the acidic-hydrophobic region of AD2 in Nrf1 around membranes.

The putative inhibition of Nrf1^(1-7)xN/D^ by glycosylation of N^300^ (Fig. 2A, *columns 2 vs 3*) was replaced by significant increases in the activity of Nrf1^N319;N/Dx6^, Nrf1^N331;N/Dx6^ and Nrf1^(4-7)xN/D^ (Fig. 2A, *columns 4, 5 and 8*). These data indicate that NST-mediated transactivation by Nrf1 is monitored by distinct glycosylation/deglycosylation status of N^300^, N^319^ and/or N^331^ (Fig. 2B, *lanes 4, 5 and 8*); glycosylation of N^319^ and/or N^331^ and their putative concomitant deglycosylation may contribute to the transactivation activity, at least, in the context of the latter three N/D mutants, and their contributions appear to be unaffected by glycosylation of N^300^ (particularly in Nrf1^(4-7)xN/D^) (*c.f. columns and lanes 4, 5 vs 8*). Similarly, the putative inhibition of Nrf1^(1-7)xN/D^ by glycosylation of N^394^/N^398^ (situated immediately to the C-terminal side of TMi) (Fig. 2A, *columns 2 vs 7*) was partially rescued by glycosylation of N^371^/N^376^ (adjoining the N-terminal side of TMi) in Nrf1^N371:376;N/Dx5^ or Nrf1^(1-3)xN/D^ (Fig. 2B, *lanes 6,9*); when compared to wild-type Nrf1, both mutants exhibited an approximately 2-fold increase in transactivation activity (Fig. 2A *columns 6,9*), though it was 30% lower than that of Nrf1^(1-7)xN/D^. The discrepant results from glycosylation at N^371^/N^376^ and N^394^/N^398^ (Fig. 2B, *lanes 6 vs 7*) indicate that the transactivation activity of Nrf1 may also be regulated through mechanisms other than glycosylation and/or deglycosylation. In addition, following PNGase digestion the wild-type 120-kDa Nrf1 glycoprotein migrated in NuPAGE gels with an apparent molecular weight of 95-kDa, which though similar to that of Nrf1^(1-7)xN/Q^, appears to be slightly faster than that of Nrf1^(1-7)xN/D^ (Fig. 2, B and C), suggesting that the N/D-mutants may be subject to additional modifications.

### Regulation of Nrf1 activity by its N-linked glycosylation status

Inhibition of N-linked glycosylation by tunicamycin results in the wild-type Nrf1 protein being expressed as a non-glycosylated 95-kDa polypeptide, with a transactivation activity that is 75% of that of vehicle-treated Nrf1 [6]. By contrast, the PNGase inhibitors Compound 24 and Compound 45, as well as Z-VAD-fmk (that block deglycosylation of N-linked glycoprotein [29], [31], [50], and see Fig. S2) increased the abundance of the 120-kDa Nrf1 glycoprotein (Fig. 2F), but decreased its transactivation activity (Fig. 2E). We therefore examined the impact that failure to glycosylate Nrf1 might have on its activity by asparagine-to-glutamine (N/Q) scanning mutagenesis across the NST domain. When compared with the deglycosylated wild-type 95-kDa protein, Nrf1^(1-7)xN/Q^ (in which all seven Asn-glycosylation sites were mutated into Gln residues that cannot be glycosylated) showed no change in its electrophoretic mobility (Fig. 2C, *lanes 11* vs *10*), though other N/Q mutants displayed relatively slower electrophoretic mobilities that varied according to the number (i.e. one to four) of the native Asn-glycosylation consensus sites that were retained in the mutant protein (*lanes 12 to 19*). Deglycosylation of these N/Q mutant proteins by PNGase F increased their electrophoretic mobilities. These results demonstrate that seven Asn consensus sites within the NST domain of Nrf1 were modified by glycosylation to a greater or less extent, and we therefore assume they were translocated into the lumen of the ER to allow post-translational modification.

Further examination of N/Q mutants demonstrated that the non-glycosylated Nrf1^(1-7)xN/Q^ mutant could transactivate ARE-driven luciferase reporter gene activity, but its activity was 70% of that of the wild-type protein (Fig. 2D, *columns 11 vs 10*), and is only 25% of that of Nrf1^(1-7)xN/D^ (Fig. 2A, *column 2*). This decrease in the activity of Nrf1^(1-7)xN/Q^ was replaced by significant increases in the activity of Nrf1^N300:N/Qx6^ and Nrf1^(4-7)xN/Q^ (Fig. 2D, *columns 11 vs 12, 18*), but not by either Nrf1^N319:N/Qx6^ or Nrf1^N331:N/Qx6^ (Fig. 2D, *columns 11 vs 13,14*). These data indicate that glycosylation and putative deglycosylation of N^300^, but not those of N^319^ or N^331^, contribute to transactivation by N/Q mutants of Nrf1 (Fig. 2C, *lanes 12 vs 13, 14*); by comparison, they appeared to make different contributions to the activity of Nrf1 N/D mutants (Fig. 2A, *columns 4, 5 and 8*). Conversely, the ability of Nrf1 to transactivate a reporter gene was substantially restricted by glycosylation of N^319^ and N^331^ (Fig. 2D, *columns 13, 14*), demonstrating that glycosylation of N^319^ or N^331^ contributes to the negative regulation of Nrf1. Moreover, comparison with the wild-type factor revealed that Nrf1^(1-3)xN/Q^ possessed an increased activity to transactivate an ARE-driven reporter gene (*columns 19 vs 10*). However, the increased activity of Nrf1^(1-3)xN/Q^ was significantly suppressed by glycosylation of N^371^, N^376^ or N^394^/N^398^ (in Nrf1^N371:N/Qx6^, Nrf1^N376:N/Qx6^ and Nrf1^N394:N398;N/Qx6^, respectively; *columns 15, 16, 17 vs 19*). Taken together, such variation in transactivation activity suggested that differential regulation of Nrf1 might be attributed to variable vectorial processes dependent on different mutant contexts, in which glycosylation and deglycosylation at N^371^/N^376^ and N^394^/N^398^ (within and around the TMi), as well as at N^300^ (located immediately to Neh5L), may elicit distinct position-dependent effects on the transcriptional activity possibly through a membrane-based mechanism within different surroundings. The possibility that deglycosylation of Nrf1 is responsible for its transactivation activity is supported by the finding that its ability to mediate ARE-driven gene expression was decreased approximately 30% to 40% (Fig. 2E) by two chitobiose-based PNGase inhibitors Compound 24 (C24) and Compound 45 (C45) [30], as well as by Z-VAD-fmk, a dual inhibitor of PNGases [49] and caspases [51], [52].

Intriguingly, treatment of COS-1 cells with chitobiose-based PNGase inhibitors caused an apparent increase in abundance of the 120-kDa Nrf1; this was accompanied by a slight enhancement in the 95-kDa protein (Fig. 2F). By contrast, the dual inhibitor Z-VAD-fmk treatment resulted in a marked increase in the abundance of either 120-kDa or 95-kDa proteins. Together with our data reported recently [29], these results indicate that the increase in the 120-kDa Nrf1 glycoprotein may be attributable to promotion of its stability by the presence of the N-glycan moiety (enabling it to be protected within the ER lumen against digestion by a cytosolic protease), and inhibition by Z-VAD-fmk of putative caspase-mediated proteolysis. The inhibition of possible proteolysis could also contribute to accumulation of the 95-kDa Nrf1 electrophoretic band; much of the Nrf1 protein in this band is likely to be the unglycosylated 95-kDa form (exhibiting a weak activity), but not the active deglycosylated 95-kDa Nrf1. This is due to the fact that the unglycosylated 95-kDa protein, rather than the deglycosylated 95-kDa protein, is predominantly expressed in PNGase inhibitor-treated cells under conditions of glucose deprivation.

### Glucose deprivation activates Nrf1 through TADs other than the NST domain

**Figure 3:**
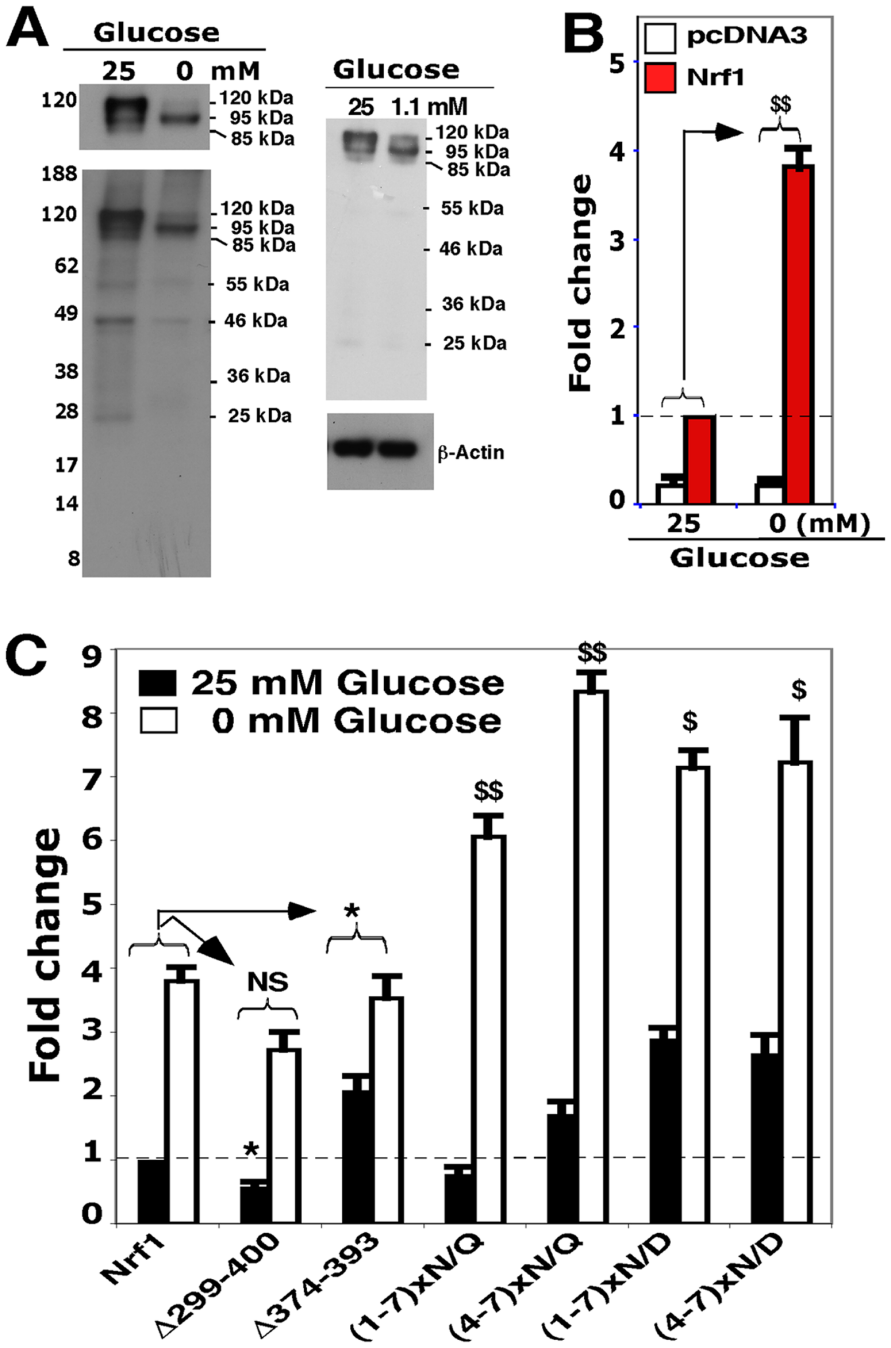
Glucose deprivation activates Nrf1 through TADs other than the NST domain. (**A**) Cells expressing wild-type Nrf1 were allowed to recover from transfection in fresh 5.5 mM-glucose-containing-medium for 8 h, and were thereafter cultured for a further 18 h in media containing 0, 1.1 or 25 mM glucose. The cell lysates were resolved by 4-12% LDS/NuPAGE, followed by immunoblotting with V5 antibodies to detect ectopic Nrf1 protein. (**B**) Increased activity of ectopic wild-type Nrf1 resulting from exposure to glucose deprivation (i.e. ‘no-glucose’) conditions ($$, p<0.001, n = 9) was determined by reporter gene assays, in which the transfected cells were allowed to recover for 8 h in medium containing 5.5 mM glucose before they were subjected to an additional 18-h culture in either glucose-free or 25-mM glucose medium. (**C**) Transactivation of an ARE-driven luciferase gene by Nrf1 or mutants, following 18-h no-glucose starvation, was calculated from three independent reporter gene assays. Significant increases in transactivation activity ($, p<0.05; $$, p<0.001, n = 9) and significant decreases (*, p<0.05; **, p<0.001, n = 9) are shown.

As the Nrf1 N/D-mutants (representing deglycosylated protein) were associated with increased transactivation of ARE-driven gene expression, we wondered whether glucose levels might alter Nrf1 activity. Western blotting showed that in cells subjected to glucose deprivation, produced by placing them in medium lacking glucose (Fig. 3A, *left*) or containing 1.1 mM glucose (*right*), Nrf1 migrated as a major 95-kDa deglycosylated protein, along with a minor 120-kDa glycosylated isoform; this was accompanied by an approximate 3.8-fold increase in Nrf1 activity when subject to growth in medium lacking glucose (Fig. 3B). However, the increase in Nrf1 activity caused by glucose deprivation could not be completely attributed to changes in its glycosylation status and/or subsequent putative deglycosylation because a significant increase in transactivation activity was also observed when Nrf1^(1-7)×N/Q^, Nrf1^(1-7)×N/D^, as well as Nrf1^(4-7)×N/Q^ or Nrf1^(4-7)×N/D^, were exposed to these conditions (Fig. 3C). Deletion of the TMi region (to yield Nrf1^Δ374-393^) increased the basal activity of Nrf1, but did not further elevate its stimulation upon exposure to glucose-free conditions. By contrast, loss of the entire NST domain (to create Nrf1^Δ299-400^) blunted both the basal activity of Nrf1 and its stimulation by glucose deprivation (Fig. 3C). These results suggest that TADs other than NST, such as AD1 and AD2, are required for the increase in Nrf1 activity during glucose deprivation.

### AD1 contributes to the transactivation by Nrf1 of its target genes

**Figure 4:**
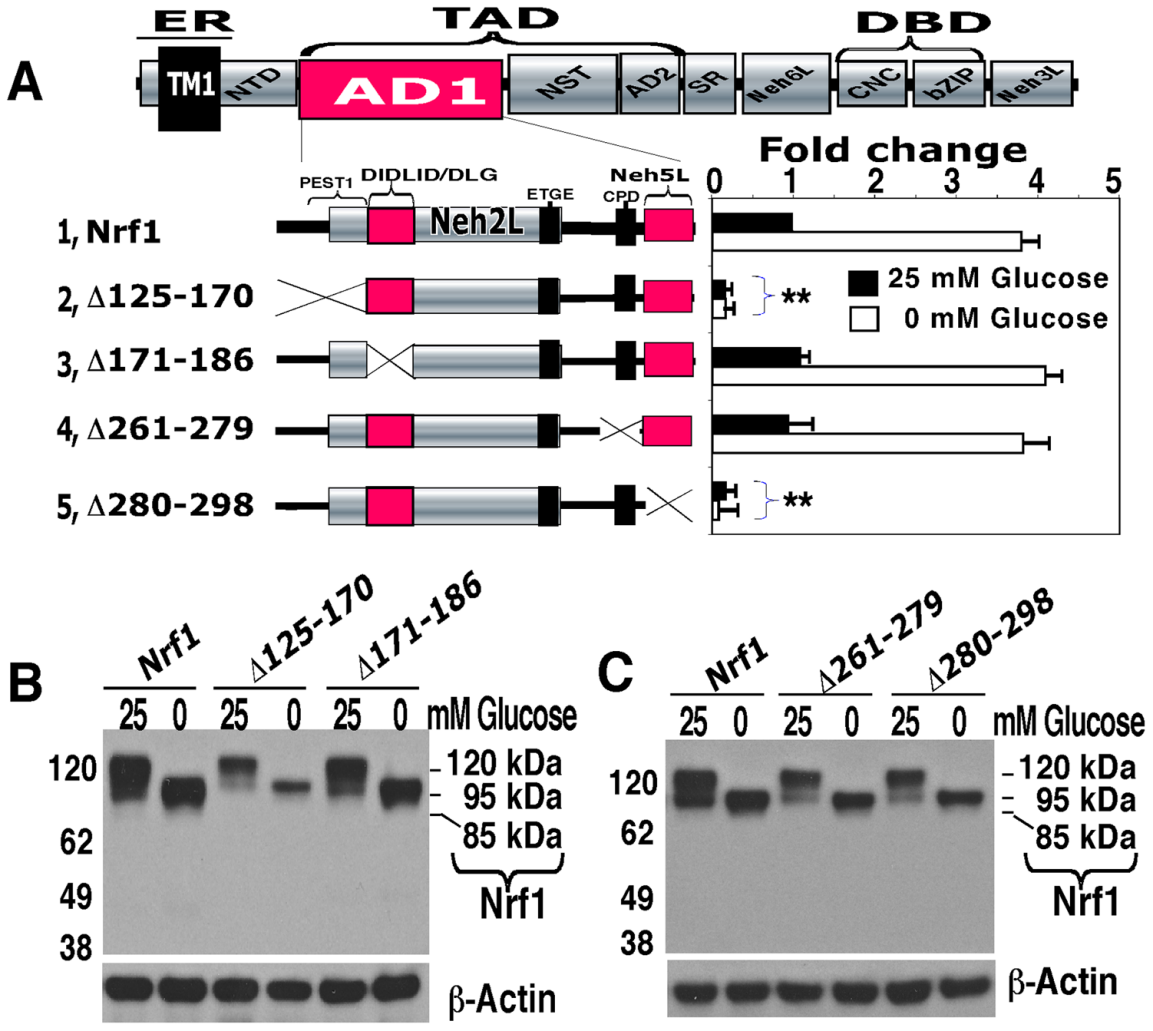
AD1 contributes to Nrf1-mediated transactivation of ARE-driven reporter genes. (**A**) The *left* schematic illustrates the relative positions of PEST1, Neh2L, CPD and Neh5L within AD1. The DIDLID/DLG element and the ETGE motif are situated in Neh2L, which overlaps PEST1. The *right* panel shows that discrete regions of AD2 make different contributions to Nrf1 activity. Cells were transfected with the indicated expression plasmids, along with that for *GSTA2-*6×ARE-Luc reporter construct. After recovery in 5.5 mM-glucose medium, the cells were cultured for a further 18 h in glucose-free or 25 mM-glucose-containing medium, before luciferase activity was measured. Significant decreases (**p<0.001, n = 9) relative to wild-type Nrf1 activity are indicated. (**B** and **C**) These samples were also subjected to western blotting and cross-reacting polypeptides were visualized by ECL.

Examination of deletion mutants lacking portions of AD1 (aa 125–298) revealed that Nrf1 requires the presence of a potential PEST1-containing sequence (aa 125–170) and Neh5L (aa 280–298) for both its basal activity and its stimulation upon glucose deprivation (Fig. 4A, *columns 2, 5 vs 1*). By contrast, neither the DIDLID/DLG element (aa 171–186) nor the Cdc4 phosphodegron (CPD, ^267^LLSPLLT^273^)-linker region (aa 261–279, situated between Neh2L and Neh5L) appeared to be required for its basal activity or its stimulation by glucose deprivation (*columns 3, 4 vs 2*). Western blotting showed that the abundance of Nrf1^Δ125-170^ and Nrf1^Δ280-298^ was decreased when compared with the wild-type protein (Fig. 4B), but their levels were enhanced by MG132 (data not shown), suggesting that the PEST1 sequence and Neh5L region may also contribute to Nrf1 stability. In addition, a decrease in the expression of Nrf1^Δ261-279^, but not Nrf1^Δ171-186^ (Fig. 4C), indicates that the CPD rather than the DIDLID/DLG element contributes to the stability of Nrf1, particularly its 120-kDa glycoprotein.

### AD1 is transiently translocated in the ER lumen before Nrf1 transactivates its target genes

**Figure 5:**
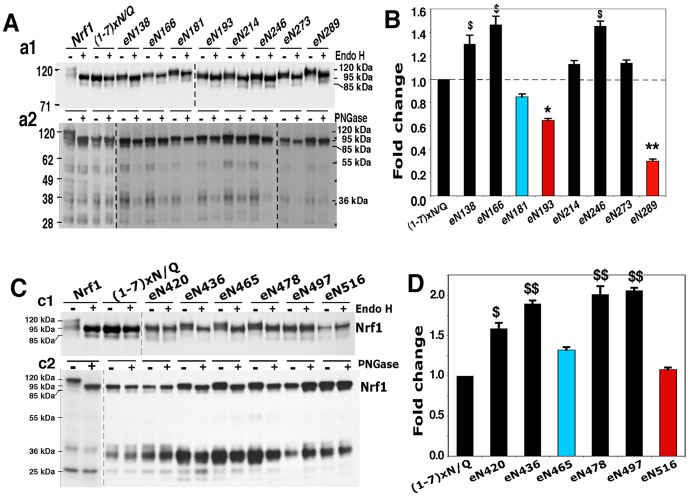
TADs are transiently translocated in the lumen of ER before transactivating Nrf1-target genes. (**A**) AD1 was mapped by the introduction of eN glycosylation sites into Nrf1^(1-7)xN/Q^ (Figure S3, A to C). Following treatment of cell lysates that expressed Nrf1 eN mutants with Endo H or PNGase F to deglycosylate proteins, the products were analyzed by LDS/NuPAGE containing 7% Tris-Acetate gel (***a1***) or 4–12% Bis-Tris gel (***a2***), before immunoblotting. (**B**) The activity of Nrf1^(1-7)xN/Q^ and its eN mutants was determined using the *GSTA2-*6×ARE-Luc reporter. Significant increases ($, p<0.05, n = 9) and significant decreases (*, p<0.05; **, p<0.001, n = 9) in the transactivation activity are shown. (**C**) Total lysates of cells expressing Nrf1 eN mutants within AD2 and SR-PEST2 (Figure S3D) were deglycosylated by digestion with Endo H (***c1***) or PNGase F (***c2***). The electrophoretic mobilities of Nrf1 proteins were monitored by immunoblotting. (**D**) The activity of Nrf1^(1-7)xN/Q^ and its eN mutants was determined using a *GSTA2-*6×ARE-Luc reporter assay. The statistical significance of data was calculated ($, p<0.05 and $$, p<0.001, n = 9).

In order to determine why the PEST1 and CPD regions exert distinct effects on Nrf1, we examined whether both sequences are translocated into the lumen of the ER. For these experiments glycosylation mapping of AD1 was performed on the unglycosylatable Nrf1^(1-7)xN/Q^ protein. We then engineered glycosylation asparagines (eN) into the AD1 of Nrf1^(1-7)xN/Q^ (Fig. S3, A to C) so that if AD1 in the mutant protein was translocated into the ER lumen, it could be glycosylated by the *in vivo* addition of glycan to the newly introduced site. Following deglycosylation by *in vitro* digestion with Endo H, each of the eight eN mutant proteins exhibited a faster electrophoretic mobility than the non-Endo H-digested proteins (Fig. 5A1, *upper*). By contrast, deglycosylation reactions with PNGase F produced electrophoretic changes in Nrf1^eN138^, Nrf1^eN214^, Nrf1^eN246^, and Nrf1^eN273^ (Fig. 5A2, *lower*). A relatively small shift in the electrophoretic mobility of Nrf1^eN181^ and Nrf1^eN289^ was observed, but not in Nrf1^eN166^ or Nrf1^eN193^ (Fig. 5A2, *lower*). These disparities in the mobility of proteins in the two deglycosylation reactions suggest that those PNGase cleavage sites between the GlcNAc residue of N-glycans and the amide group of glycosylated Asn residues 166, 181, 193 or 289 (around the DIDLID/DLG element and Neh5L region) may be buried in an incompletely-denatured or partially-recovered conformation after denaturation of Nrf1, as described for other membrane proteins [53], [54].

Whilst the entire AD1 region translocates into the ER lumen, those peptide sequences around the DIDLID/DLG element and the Neh5L subdomain are likely to be folded (as wheeled in Fig. S1C) in close proximity to membranes because this would enable an adjacent potential cholesterol recognition amino acid consensus motif (CRAC3, L/V-x_1-5_-Y-x_1-5_-R/K [55], Fig. S4B) in Nrf1 to interact with membrane lipids. This interpretation is supported by our finding that the transactivation activity of Nrf1^(1-7)xN/Q^ is significantly decreased by glycosylation of eN^289^ (within the Neh5L) (Fig. 5B), and is also partially inhibited by glycosylation of eN^193^ (within CRAC3 immediately to the DIDLID/DLG element). By contrast, glycosylation of eN^181^ (within the DIDLID/DLG element) resulted in the activity of Nrf1^eN181^ to be blunted by 15% of that of the wild-type factor. Conversely, glycosylation of eN^138^, eN^166^ (both situated within the PEST1 sequence), and eN^246^ (between the CPD and Neh2L regions) caused a significant increase in the reporter activity (Fig. 5B). These results suggest that the position-dependent glycosylation of Nrf1 might affect its topological folding in distinct vectorial processes, as has been described for other membrane glycoproteins [56], [57].

### The DIDLID/DLG element, Neh2L and Neh5L regions differentially affect the repartitioning of AD1 from the ER luminal side of membranes into the cyto/nucleoplasmic compartments

**Figure 6:**
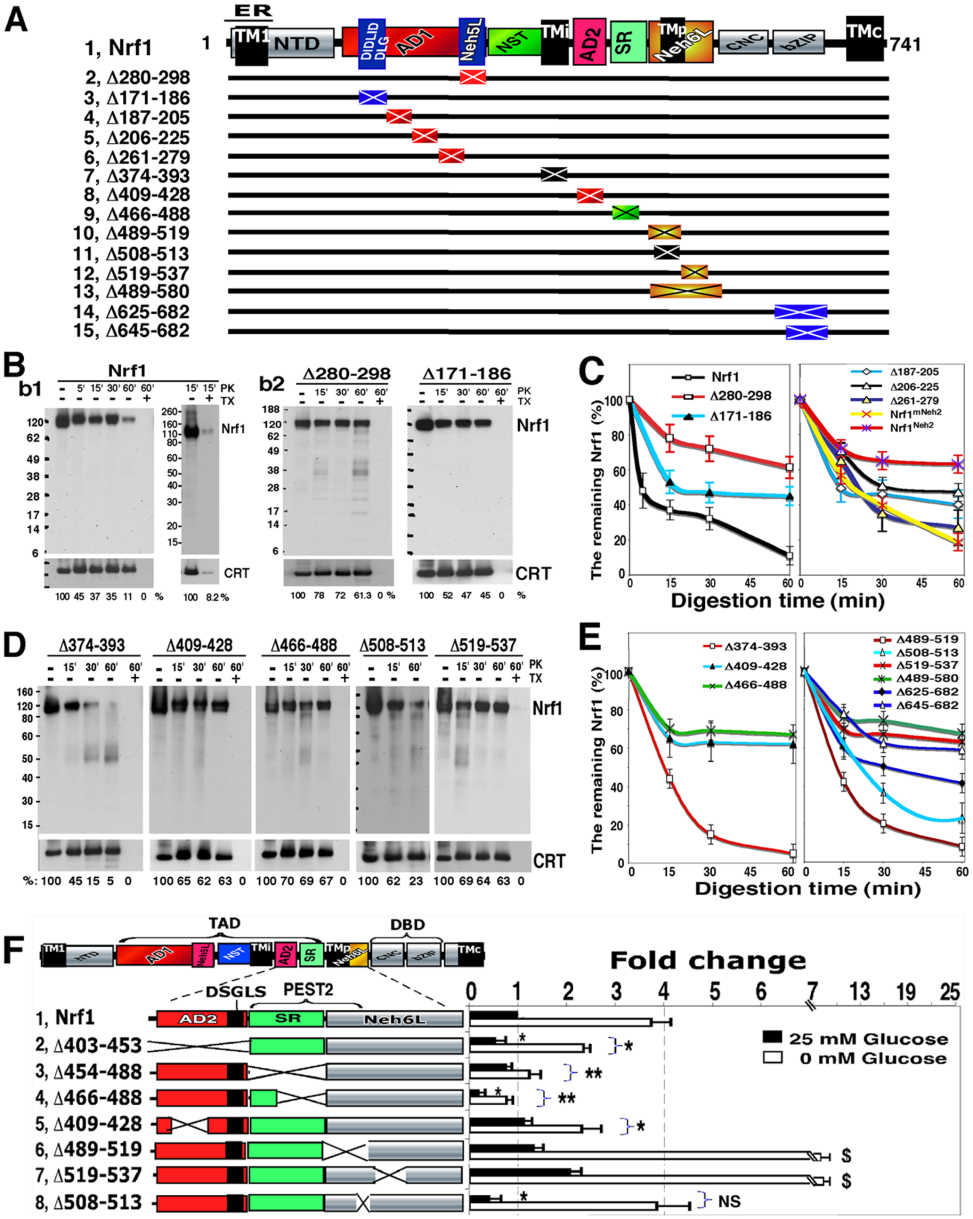
Partial repartitioning of the NST-adjoining TADs across membranes into the cyto/nucleoplasm. (**A**) Schematic of a series of Nrf1 deletion mutants lacking discrete portions of AD1 (including Neh5L and DIDLID/DLG), TMi-containing NST, AD2, SR, TMp-containing Neh6L, and bZIP. In addition, the locations of the eN mutants are also indicated across the AD2, SR and Neh6L domains. (**B** and **C**) Cells expressing wild-type Nrf1 (***b1***), its mutant Nrf1^Δ280-298^, Nrf1^Δ171-186^ (***b2*** **)**, or others indicated (***C***) were subjected to subcellular fractionation, followed immediately by an intact ER membrane protection assay to measure the sensitivity of the ectopic proteins to digestion by PK (50 μg/ml); proteolysis was allowed to proceed in the presence or absence of 1% TX in reaction mixtures placed on ice. The products were examined by immunoblotting with polyclonal antibodies against Nrf1β before being re-probed with antibodies against calreticulin (CRT) as a marker for luminal proteins. The intensity of these blots was estimated by dividing the value for Nrf1 with that for CRT, and the relative percentage (%) amount of Nrf1 that remained after PK digestion was normalized to the total amount of Nrf1 in reactions without PK digestion. The results are shown graphically (*c,* mean ± S.D, n = 4), allowing the stability of different Nrf1 mutants in membrane PK protection reactions to be compared (see Figure S4). (**D** and **E**) Membrane PK protection reactions using intact ER-enriched fractions purified from cells expressing Nrf1^Δ374-393^, Nrf1^Δ409-428^, Nrf1^Δ466-488^, Nrf1^Δ508-513^ or Nrf1^Δ519-537^ proteins (***D***) or other mutants indicated (***E***). The relative percentage of protein remaining after PK digestion was calculated as described above. The results are shown graphically (*e,* mean ± S.D, n = 4), allowing the stability of different Nrf1 mutants in membrane PK protection reactions to be compared (also see Figure S6). (**F**) The *left* schematic shows Nrf1 mutants lacking various portions of the protein. Their contributions to changes in Nrf1 activity in response to glucose starvation, when compared with activity observed under 25 mM-glucose conditions (control), were examined using the reporter assay. Significant increases ($, p<0.05 and $$, p<0.001, n = 9) and decreases (*p<0.05, **p<0.001, n = 9) are indicated, relatively to the wild-type Nrf1 activity obtained from the 25 mM-glucose conditions.

**Figure 7:**
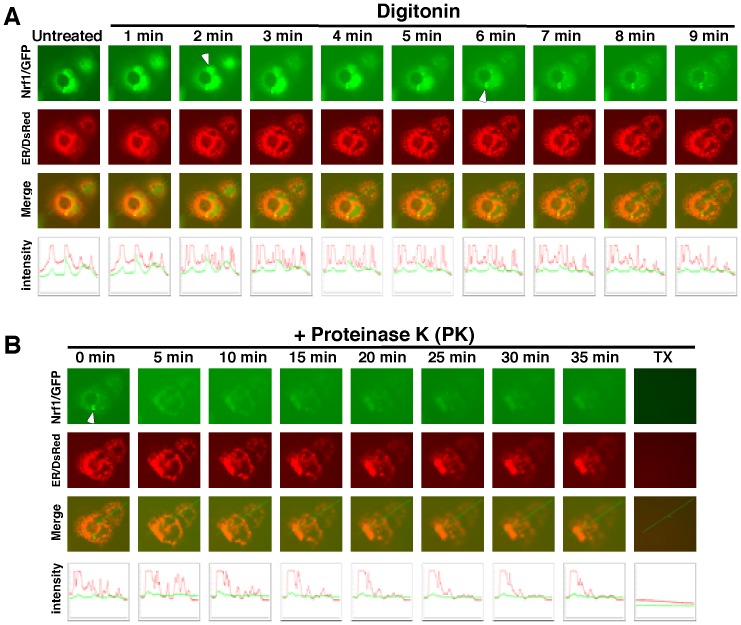
Live-cell imaging of Nrf1/GFP to determine its dynamic movement out of the ER into the cytoplasm. COS-1 cells were co-transfected with expression constructs for Nrf1/GFP fusion protein and the ER/DsRed marker, and were then subjected to live-cell imaging combined with the *in vivo* membrane protease protection assay. (**A**) The cells were permeabilized by digitonin 20 mg/ml) for 10 min, (**B**) before being co-incubated with PK (50 mg/ml) for 35 min prior to addition of 1% Triton X-100. Over this time interval, real-time images were acquired using the Leica DMI-6000 microscopy system. The merged images of Nrf1/GFP with ER/DsRed are presented (on *the third row of panels*), whereas changes in the intensity of their signals are shown graphically (*bottom*). The characteristic features of the arrow-indicated cells are described in the main text. Overall, the images shown herein are a representative of at least three independent experiments undertaken on separate occasions that were each performed in triplicate (n = 9).

Movement of Nrf1 from the ER lumen into the cyto/nucleoplasm was assessed using time-course membrane proteinase protection reactions with PK. As shown in Figure 6B1, 60% and 90% of the 120-kDa Nrf1 glycoprotein was proteolytically degraded by PK following 15 and 60 min incubation, respectively, of intact ER-rich membranes (*left*). By contrast, inclusion of 1% TX in reactions, to solubilize membranes, led to the disappearance of essentially all Nrf1 protein by 15 min (*right*). Loss of Nrf1 protein in digests that lacked TX demonstrated that a ∼30% fraction of the NHB1-CNC protein is susceptible to proteolysis even in the presence of membranes, suggesting that a portion is dynamically repartitioned across membranes into the extra-luminal side. This conclusion is also supported by live-cell imaging of Nrf1-GFP fusion proteins (Fig. 7). In addition, the remaining ∼10% Nrf1 proteins following 60-min incubation with PK would be completely digested and would disappear as its dose was increased or its incubation time was extended ([6], [29] and data not shown).

Comparison of Nrf1 with its mutants in membrane protection assays revealed that putative retrotranslocation of Nrf1 across membranes was largely blocked upon deletion of its Neh5L subdomain (in Nrf1^Δ280-298^, Fig. 6B2); interestingly, this was associated with a marked loss of transactivation activity (Fig. 4A). Additional protection against degradation in the membrane PK reactions was observed upon deletion of either the DIDLID/DLG element (in Nrf1^Δ171-186^, Fig. 6B2) or disruption of a predicted α-helix formed by aa 194-226 within Neh2L (in Nrf1^Δ206-225^, Figs. 6C and S4F). By contrast, the protection of Nrf1 by membranes against PK was unaffected by the loss of CPD in Nrf1^Δ261-279^ (Fig. S4F). Collectively, these results suggest that the AD1, along with the NST glycodomain, is reintegrated into the ER, and then dynamically repartitioned out of membranes into the cyto/nucleoplasmic compartments probably through Neh5L and DIDLID/DLG.

Next we examined whether the DIDLID/DLG element within Neh2L (aa 156-242) influences the partitioning of AD1 around membranes by using a chimaeric Nrf1^Neh2^ protein (in which its Neh2L region was replaced by the Neh2 domain of Nrf2, Fig. S4A) and the substituted Nrf1^mNeh2^ (in which mNeh2 represents a mimicked form of Neh2 where the DLG and ETGE motifs of Nrf1 were substituted with those in Nrf2 (Fig. S4B). Membrane proteinase protection reactions revealed that Nrf1^Neh2^ exhibited behaviour similar to that of Nrf1^Δ280-298^ (Figs. 6C and S4D), but the repartitioning of Nrf1 around membranes appeared to be unaltered in Nrf1^mNeh2^. The different sensitivities of Nrf1^Neh2^ and Nrf1^mNeh^ to proteinase digestion appeared to result from the presence of a potential CRAC3 motif in Neh2L and mNeh2, that is absent from the Neh2 domain of Nrf2 (Fig. S4B). This conclusion is supported by data showing that loss of CRAC3 partially enhanced membrane protection of Nrf1^Δ187-205^ against PK digestion (Figs. 6C, and S4F).

The repartitioning of the CRAC3-adjoining DIDLID/DLG element out of membranes was explored further by using the triple sandwich fusion protein DsRed/N275/GFP (where N275 represents the N-terminal 275 amino acids of Nrf1) in a membrane PK protection assay (Fig. S5A). Immunoblotting with antibodies against DsRed or GFP revealed that the DsRed/N275/GFP fusion protein of 85 kDa integrated into the ER and nuclear membranes, and that DsRed was degraded by PK more quickly than was GFP (Fig. S5B). These data demonstrate that the DsRed epitope was positioned on the cyto/nucleoplasmic side where it is not protected by membranes. By contrast, the N275 polypeptide fused to GFP was dynamically repartitioned from the ER luminal side across membranes (through an unknown mechanism) into the cyto/nucleoplasmic compartment (Fig. S5D), where it was digested upon exposure to PK to yield multiple digested polypeptides of between 25 kDa and 65 kDa (Fig. S5C). The abundance of a major 30-kDa GFP fusion polypeptide also gradually decreased with PK digestion alone as the incubation time increased, but it was not completely eliminated by PK, even in the presence of TX. We also noted that the ER rather than nuclear membranes appeared resistant to TX (Fig. S5C), suggesting that Nrf1 may associate with a detergent-resistant microdomain within the ER.

### The AD2, SR and Neh6L regions make distinct contributions to the partial repartitioning of Nrf1 across membranes into the cyto/nucleoplasmic side

Membrane proteinase protection assays showed that Nrf1^Δ374-393^ (lacking the TMi region located immediately to the N-terminal side of AD2) was almost completely digested after 30-min incubation with PK, but that Nrf1^Δ409-428^ (lacking much of the acidic-hydrophobic amphipathic portion of AD2) and Nrf1^Δ466-488^ (lacking much of SR C-terminally to AD2) were largely protected by membranes against PK digestion (Fig. 6, D and E). These findings indicate that the TMi glycopeptide serves as a luminal anchor to restrict the repartitioning of Nrf1 out of ER membranes, whilst both the amphipathic AD2 region and the SR domain promote the transfer of Nrf1 from the lumen out of membranes.

Further, glycosylation mapping of AD2, SR and PEST2 within Nrf1^(1-7)xN/Q^ revealed that eN^436^, eN^465^ and eN^478^ were transiently partitioned into the lumen of the ER where they were glycosylated (Fig. 5C), whereas non-glycosylated eN^420^ (in the amphipathic portion of AD2), and eN^497^ and eN^516^ (both in the C-terminal border between SR and Neh6L) could be either integrated within membranes or positioned in the extra-luminal subcellular compartments (Fig. 5C). Surprisingly, the transcriptional activity of none of the six Nrf1^eN^ mutants was diminished by the introduction of glycosylation sites, Instead, the eN^420^, eN^436^, eN^478^ and eN^497^ mutants exhibited a significant increase in the reporter activity (Fig. 5D), suggesting that these eN-adjoining portions of AD2 and SR contribute to the positive regulation of Nrf1. Together with bioinformatic analyses (Fig. S1B), these results suggested that glycosylated Asn residues around the TMi peptide do not serve as a stable integral transmembrane determinant. Rather, the core Phe/Leu-rich region (aa 375-393) probably serves as a membrane-tethered determinant because it lies topologically on the plane of the luminal leaflet of membrane lipid bilayer, and anchors the adjoining amphipathic portion of AD2 close to the luminal interface of membranes. In this case, once the TMi glycopeptide is deglycosylated, its adjacent regions (i.e. AD1, AD2 and SR) should be liberated from the luminal confinement, and repartitioned out of membranes into cyto/nucleoplasmic compartments enabling transactivation of Nrf1-target genes. This assumption is supported by the observation that the basal Nrf1 activity and/or its stimulation by glucose deprivation were, to varying degrees, prevented by deletion of the entire AD2, SR domain or their major portions (Fig. 6F).

By contrast with AD2, discrete portions of the Neh6L domain seem to be located in extra-luminal (i.e. in juxtamembrane, intramembrane, or cyto/nucleoplasm) subcellular compartments. Weight is given to this conclusion by the mapping data showing that neither eN^497^ nor eN^516^, in the N-terminal portion of Neh6L that overlaps the PEST2 sequence, are glycosylated (Fig. 5C). Moreover, N-linked glycosylation of Nrf1 within the ER lumen occurs at its seven Asn residues that reside in the NST domain, but not at the N^543^ glycosylation consensus ^543^ NHTY^546^ motif within the Neh6L domain (Fig. S6A). These observations, together with bioinformatic analyses and membrane protection assays (Figs. 6D and S6), led us to envisage that an N_lum_/C_cyt_-orientated transmembrane region in Nrf1 exists between aa 497 and 543. Although this region does not possess sufficient hydrophobicity to fold into a single stable helix that spans the membrane, it may be able to do so through intramolecular and/or intermolecular interactions with other protein helices within membranes, as described elsewhere [58], [59]. Such an interaction may drive the folding of the semihydrophobic amphipathic peptide ^507^AEGAVGYQPEYSKFCRMSY^525^ (containing a CRAC-adjoining transmembrane helix-helix interaction motif, Fig. S1B) to enable the formation of a putative proline-kinked hinge structure (TMp), with the topology of its net positively-charged semihydrophobic region lying on the interface of membranes or spanning membranes.

To gain an insight into the biological significance of the supposed TMp region, we examined the consequence of disruption of TMp-adjoining peptides. The resulting mutants exhibited strikingly different sensitivities to PK digestion in the membrane protection assay (Fig. 6E). Deletion of the N-terminal TMp hexapeptide ^508^EGAVGY^513^ caused 23% of Nrf1^Δ508-513^ to be protected by membranes after 60-min incubation with PK (Fig. 6D), and also resulted in a reduction in transactivation activity to 45% of that of wild-type Nrf1 under homeostatic conditions (Fig. 6F). By contrast, Nrf1^Δ489-519^ (lacking the ^507^AEGAVGYQPEYSK^519^ core TMp region along with its N-terminally flanking negative SDS2 peptide) was not protected by membranes against PK digestion (Figs. 6E and S6B), indicating that its repartitioning from the lumen out of membranes is enhanced. Conversely, Nrf1^Δ519-537^ (lacking the positively charged ^519^ KFCRMSY^525^ and its C-terminally flanking peptide) was largely protected by membranes against PK digestion (Fig. 6, D and E). These findings are consistent with the idea that this positive region enables the post-insertion of TMp into membranes and its topological orientation to be determined, according to the positive-inside rule [1], [58]. It is therefore assumed that the C-terminally TMp-flanking portions of Nrf1 are allowed to position on the cyto/nucleoplasmic side of membranes. This interpretation is also supported by membrane protection assays in which Nrf1^Δ489-580^ (lacking the Neh6L domain) and Nrf1^Δ625-682^ (lacking the bZIP domain) appeared relatively resistant to PK (Figs. 6E and S6C). Intriguingly, although the major portion of 120-kDa Nrf1^Δ519-537^ and Nrf1^Δ489-580^ were retained in the lumen (Figs. 6D and S6C), both mutants were still processed to yield the 95-kDa and 85-kDa proteins, rather than the 36-kDa isoform (data not shown), and the presence of the 95-kDa and 85-kDa proteins was accompanied by an approximately 2-fold increase in transactivation activity under both basal and glucose deprivation-stimulated conditions (Fig. 6F). Collectively, these findings indicate that the TMp-adjoining regions may be associated with membranes and may also contribute to the negative regulation of Nrf1 by its Neh6L domain.

### Movement of Nrf1/GFP from the ER luminal side of membranes into the cyto/nucleoplasm side enables it to be proteolytically digested by proteases

The above-described results, together with our recent work [6], [29], indicate that Nrf1 is a membrane-protein that can adopt dynamic membrane-topologies that are determined by TM1. To test this hypothesis, we performed live-cell imaging of Nrf1/GFP combined with *in vivo* membrane protease protection assays, in order to determine whether it is capable of being moved from the lumen of the ER to the cyto/nucleoplasmic side of the membrane, whereupon it would become vulnerable to digestion by PK because membranes would no longer afford protection against proteolysis. As anticipated, the green fluorescent signal from Nrf1/GFP appeared to be located primarily in the ER-surrounding subcellular compartments, because the images were superimposed with red fluorescent networks presented by ER/DsRed (Fig. 7A). Upon exposure of the cells to digitonin for 2 min, a ‘hernia-like’ vesicle was observed that protruded from the cytoplasm (indicated by *arrow*) and subsequently this convex structure disappeared after 5 min incubation with digitonin. Thereafter the remaining Nrf1/GFP images became weaker, although they resembled those of ER/DsRed. These findings indicate that a cytosolic fraction of Nrf1/GFP may diffuse across permeabilized plasma membranes, and that this fraction together with an additional cytoplasmic fraction originating from the ER lumen, may also be partially digested by proteases in the cytoplasm.

Upon exposure to PK, the cells reduced in size so that the local intensity of the ER/DsRed signal appeared to increase. As such, the ER-resident signal from Nrf1/GFP (with the C-terminus facing the ER lumen) seemed to be partially protected by the membrane against 5-min PK digestion (Fig. 7B). Subsequently, the remaining Nrf1/GFP signal slowly diminished as the PK digestion time was extended to 35 min, but the residual green fluorescent signal was retained until TX was added to disrupt the ER membrane (Fig. 7B). These observations indicate that the ER-resident Nrf1/GFP fraction can be dynamically repartitioned and retrotranslocated from the luminal side of the membrane into the cyto/nucleoplasmic compartments, where the fusion protein is vulnerable to digestion by PK, but a small fraction of the ER-resident Nrf1/GFP appears to be protected by the intact membranes.

The extra-ER Nrf1/GFP signal (i.e. in the nucleus of *the right upper cell*, Fig. 7A) gradually became weaker after 3 min of digitonin incubation until it had mostly disappeared when the time of digitonin incubation was extended to 10 min. This finding demonstrates that the extra-ER fraction of Nrf1/GFP is unlikely to be protected by the nuclear envelope membranes. In addition, we also noted that a fraction of the fusion protein could be localized within the juxtanuclear aggresome-like P-bodies around and within the ER (indicated *by arrow*), but this possibility remains to be studied in more detail.

## Discussion

In the present study we found that the post-translational modification and processing of Nrf1 is controlled by its membrane-topology. The repartitioning of Nrf1 across ER membranes into the cyto/nucleoplasmic side enables the CNC-bZIP factor to transactivate ARE-driven genes through its acidic-hydrophobic amphipathic glucose-responsive domains.

### Nrf1 is an integral membrane-spanning protein that entails dynamic membrane-topology

The biological function of the membrane-bound Nrf1 is probably largely dictated by its dynamic topological folding and its movement into and out of the ER lumen. Such events occur before Nrf1 is able to transactivate its target genes. According to current knowledge of protein folding within membranes [1], [58], [60], the topology of Nrf1 is likely to be determined by hydrophobic, semihydrophobic, amphipathic, and other topogenic signals (collectively called *topogon*) within the protein. It is thus postulated that the topogons of Nrf1 (e.g. TM1, TMi, TMp and TMc)^28^ are decoded by molecular machines (i.e. *ribosomes* and *translocons*) into an initial topological structure partitioned within and around the specific membrane microdomain. Subsequently, the membrane-topogenic folding of Nrf1 is likely to be modulated through: i) the intramolecular interactions between its own helical regions; ii) the intermolecular interactions of its helical regions with those in other membrane proteins (e.g. *retrotranslocons* and *flippase*); and/or iii) other interactions of the juxtamembrane helix-adjacent regions (e.g. CRACs) with membrane lipids (e.g. cholesterol and sphingolipids that are enriched in the detergent-resistant microdomain (i.e. lipid rafts and caveolae [58], [59]). These putative interactions, together with the topogons of Nrf1, dictate the formation of the final topological structure and position of the transcription factor within and around membranes.

Our data demonstrate that Nrf1 can adopt distinct dynamic membrane-topologies that are determined by its N-terminal TM1 sequence (that exists in an N_cyt_/C_lum_ orientation within membranes) in cooperation with other semihydrophobic amphipathic TMi, TMp and TMc regions. In fact, the TM1 peptide is not highly hydrophobic, and thus this characteristic will allow a small fraction of Nrf1 to be released into the extra-membranous cytoplasmic or luminal compartments. In turn, the luminal portion within Nrf1 may also refold and then become reintegrated into the ER membrane[29]. Furthermore, our live-cell imaging results, together with both membrane proteinase protection assays and mutagenesis analyses, demonstrate that the membrane-topologic organization of Nrf1 is highly dynamic (in particular TMi, TMp and TMc) and its orientation can also be regulated by changes in the membrane lipids and the surrounding environment. Overall, the membrane-topological processes of Nrf1, in which it is dynamically moving in and out of membranes, control both its post-translational modification to generate distinct isoforms and its transactivation activity to regulate its target gene expression.

According to the accumulating knowledge, the orientation of membrane-topology is determined by the positive-inside and charge difference rules, besides the hydrophobic gradient along membranes [1], [58]. The topogenesis of membrane-protein is also influenced by its glycosylation and/or deglycosylation status [56]. Glycosylation of the TM-adjoining peptide can allow it to be anchored within the ER, but it may also act as an unidentified signal for glycan-recognition machinery involving dislocation of proteins into the extra-ER compartments. In this vectorial process, deglycosylation of glycoprotein enables conversion of the zero-charged Asn into the negative-charged Asp residues (pKa  = 3.9), increasing the transactivation activity. Collectively, these proposed mechanisms appear to explain why N^300^ (immediately located to the Neh5L, an essential TAD element) within the contexts of Nrf1^N300;N/D×6^, Nrf1^(4-7)×N/D^ and Nrf1^N300;N/Q×6^ mutant proteins contributes to distinct and opposing variations in ARE-driven gene activity. The results reveal that only N^300^ glycosylation/deglycosylation status of Nrf1^N300;N/D×6^ is sufficient to maintain the transcription activity similar to wild-type Nrf1, whilst a combination of the intact status of N^300^, N^319^ and N^331^ in Nrf1^(4-7)×N/D^ causes an increase in the transcription activity. The higher activity of Nrf1^(4-7)×N/D^ than Nrf1^N300;N/D×6^ is thought to be due to its partial movement out of ER in the vectorial process whereby glycosylated N^319^ and N^331^, besides N^300^, are deglycosylated into Asp residues. By comparison with Nrf1^N300;N/Q×6^ that exhibits an increased transcription activity, Nrf1^N300;N/D×6^ is relatively acidic so that it is likely to reside in the oxidizing ER lumen. These intriguing findings have led us to surmise that the N^300^-directed vectorial processes of Nrf1^N300;N/Q×6^, rather than Nrf1^N300;N/D×6^, might facilitate the former mutant to dislocate from the ER into the cyto/nucleoplasm.

### Nrf1 activity is controlled by the repartitioning of its acidic transactivation domains across ER membranes that, in turn, dictates post-translational modifications of the NHB1-CNC factor

**Figure 8:**
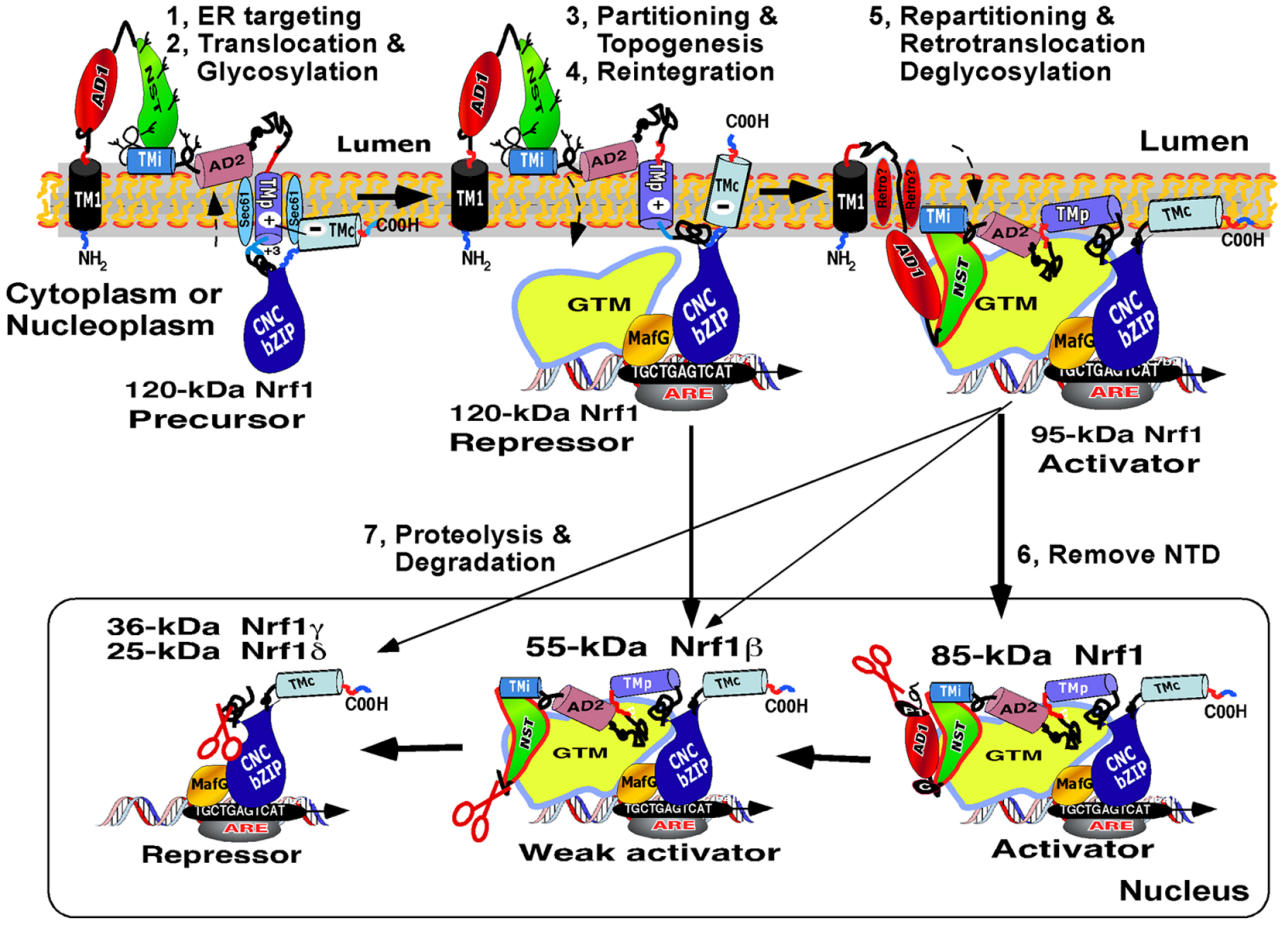
A proposed model to explain the molecular mechanisms controlling Nrf1. Since Nrf1 is a mobile membrane-associated protein that engages in dynamic topologies [29], we propose a model to explain the molecular mechanisms controlling both its post-translational processing and its activity. The model involves seven stages. **I**) After being targeted to the ER, Nrf1 is anchored in the membrane through TM1. **II**) The NST-adjoining TADs in Nrf1 are transiently translocated into the lumen, where they are glycosylated to yield a 120-kDa glycoprotein. **III**) During topogenesis, the TMi-adjacent amphipathic regions in Nrf1 are tethered to the luminal leaflet of the membrane, whilst TMp dynamically associates within membranes, and its flanking PEST2 and Neh6L may be partitioned into distinct compartments. During this stage, the basic CNC-bZIP domain is retained in the cyto/nucloplasm, and its connecting TMc region is likely to be either left in the cytoplasm or integrated into membranes. **IV**) Once the TMi region in Nrf1 is liberated from the restraint of its flanking glycopeptides, it is reintegrated into membranes. This process should enable repartitioning of AD2 and SR out of membranes enabling it to function as a TAD. **V**) When required, the luminal NST and AD1 are repartitioned across the membrane into the cyto/nucleoplasm, thereby enabling deglycosylation of Nrf1 to produce the 95-kDa active transcription factor that up-regulates genes through its TADs. **VI**) An 85-kDa cleaved isoform of Nrf1 is generated upon removal of the NTD, allowing it to be released into the nucleus where it transactivate ARE-driven genes. **VII**) Distinct degrons can trigger proteolysis of Nrf1 to yield 55-kDa Nrf1β/LCR-F1 (acting as a weak activator), and/or the dominant-negative 36-kDa Nrf1γ and 25-kDa Nrf1δ isoforms. Abbreviations: GTM, general transcriptional machineries; ‘Retro?’, an unidentified retrotranslocon complex.

The model shown in Figure 8 depicts membrane-topological mechanisms by which we propose Nrf1 is selectively activated and inactivated through dynamic repositioning within and/or around membranes. During the co-translational topogenesis of Nrf1, its AD1 (containing PEST1, Neh2L, CPD and Neh5L) and AD2, together with the NST glycodomain, are translocated into the ER lumen. This vectorial process enables insertion of the nascent 95-kDa Nrf1 polypeptide into the ER lumen, where it is glycosylated through its NST domain to yield a 120-kDa glycoprotein that is inactive because its TAD regions (including AD1, NST, AD2 and SR) are buried within the ER lumen. However, when biological cues trigger induction of Nrf1-target genes, the luminal AD1, AD2 and the NST glycodomain of the 120-kDa glycoprotein are partially repartitioned out of membranes, so that they can be dynamically retrotranslocated or dislocated across ER membranes into the cyto/nucleoplasm, allowing its deglycosylation and the generation of an active 95-kDa Nrf1 transcription factor. The topological repartitioning of Nrf1 possibly occurs through the existence of acidic-hydrophobic amphipathic regions (i.e. DIDLID/DLG, Neh5L, TMi, AD2 and SR), but it is not known which ER-to-cytosol retrotranslocon-competent proteins (e.g. Derlins and Sec6 [61], [62]) are involved in the vectorial processing of Nrf1.

Once the 120-kDa Nrf1 glycoprotein enters the cytoplasm and/or nucleoplasm, it is subject to N-linked deglycosylation by PNGase [29], before it is modified by calpain- and/or proteasome-mediated proteolysis [30], [63], [64] to yield distinct fragments of between 25-kDa and 95-kDa, each with a distinct function. It should be noted that we have yet to demonstrate that Nrf1 is enzymatically deglycosylated following its retrotranslocation from the ER lumen, but this seems to be probable. We therefore postulate that the extent to which Nrf1 is deglycosylated is also determined by the ER-associated extraction and proteasomal degradation events. This notion is based on reports that during deglycosylation of N-linked glycoprotein, PNGase enables interactions of the deglycosylated protein with the ER-to-cytosol retrotranslocation -coupled extraction machinery components (i.e. Derlin-1 and VCP/p97) through its N-terminal PUB (peptide:N-glycosidase/UBA or UBX-containing) domain [65], [66]. During this process, PNGase directly binds the ubiquitin-like domain (UBL) of HR23, allowing an engagement with the 26S proteasome [67], [68].

Besides deglycosylation, the Hrd1- and VCP/p97-dependent ERAD pathway allows proteolysis of the membrane-bound Nrf1 protein to yield a potential transcriptional activator of 85-kDa [9], [31], though the ER/NE-resident ubiquitn ligase Dao10/TEB4-directed pathway [69] cannot be ruled out. Formation of the 85-kDa isoform appears to be partially prevented by removal from the NTD of Nrf1 of aa 55–80 covering CRAC1/2[34], or by deletion of aa 31-80 (containing a putative Hrd1-binding site [64]). However, the detailed mechanisms whereby the Hrd1- and VCP/p97-dependent extraction pathways control the ER-to-nuclear retrotranslocation of Nrf1 have not been elucidated. Moreover, Hrd1- and VCP/p97 also seems to repress Nrf1 because they are involved in ERAD of the transcription factor. Alternatively, other retrotranslocon-competent extraction machineries, such as Derlins and Sec61 complexes, may control the vectorial processing of Nrf1 across membranes. It is also unknown whether the repartitioning of Nrf1 in and out of membranes is monitored by a flippase-driven mechanism, as described for other membrane proteins [70], [71].

We note that selective proteolytic processing of Nrf1 is likely to be controlled by its dynamic membrane-topologies, which allow the repositioning of several potential degrons that target the NHB1-CNC protein for proteasome- and/or calpain-mediated degradation pathways (data shown elsewhere). Neither the PEST1 sequence nor the Neh2L subdomain within AD1 function as *bona fide* degrons for Nrf1, at least when they are transiently buried within the ER lumen. Conversely, PEST1 and Neh2L contribute to the stability of Nrf1, in particular stability of the full-length 120-kDa glycoprotein. By contrast, the AD2-adjoining DSGLS motif and the Neh6L-overlapped PEST2 sequence contribute to negative regulation of Nrf1 by allowing proteasome- and/or calpain-mediated proteolytic processing to produce the 55-kDa Nrf1β/LCR-F1 (as a weak activator [6], [72]-[74], and the 36-kDa Nrf1γ, and 25-kDa Nrf1δ dominant-negative forms. The negative regulation of Nrf1 by Neh6L is attributed to the possible degrons situated immediately to the N-terminal and C-terminal ends of TMp, suggesting that TMp serves as a flexible hinge switch to control the repositioning of PEST2 to allow selective proteolytic processing of the transcription factor. In addition, others have reported that the CPD degron (situated close to Neh5L within AD1) and the DSGLS sequence within AD2 of Nrf1 are recognized by FBW7 and β-TrCP, which target the 95-kDa Nrf1 protein for Cullin-1 directed proteasomal degradation [30], [64]. Collectively, these findings suggest that selective proteolysis of Nrf1 is dependent on the positioning of cleavage sites within the protein around and within membranes.

### Concluding comments

Our present study provides a better understanding of how the membrane-topology of Nrf1 controls its function. Movement of certain regions of Nrf1 across membranes determines the extent to which its NST domain is glycosylated in the ER lumen and deglycosylated in the extra-luminal cyto/nucleoplasmic compartments, and also the extent to which the NHB1-CNC factor can interact with ARE sequences in target genes and recruit the general transcriptional machinery. Consistent with this hypothesis, we found that those Nrf1 mutants that displayed resistance to proteolysis in membrane protection assay exhibited little transactivation activity. The inverse relationship between resistance to proteolysis and transactivation of target genes indicates that Nrf1 is positively controlled by the repartitioning of regions such as AD1-Neh5L, NST-TMi, AD2 and SR, from the luminal side of membranes into the nucleus. However, we also found that those Nrf1 mutants that exhibited an increased transactivation activity either displayed sensitivity to proteolysis and/or prevented production of short dominant-negative forms. These observations suggest that the repositioning of the TADs can also allow their coupled degrons to be selected for targeting of Nrf1 to proteasome- or calpain-mediated proteolysis pathways in order to generate active or dominant-negative isoforms. Overall, our findings have provided a framework within which the regulation of NHB1-CNC subfamily proteins including Nrf3, CncC, Skn-1, and other integral membrane proteins, can be understood in terms of topogenesis, translocation, repartitioning, dislocation, glycosylation, deglycosylation, and selective proteolytic processing.

## Supporting Information

Figure S1 **Comparison of topological determinants of NHB1-CNC factors within membranes.**
(TIF)

Figure S2 **Structural differences between the chitobiose-based PNGase inhibitors and Z-VAD-FMK.**
(TIF)

Figure S3 **Engineered glycosylation mapping of AD1, AD2, SR and PEST2 within Nrf1.**
(TIF)

Figure S4 **Opposing roles for Neh2L and Neh2 in regulating the function of Nrf1** ***versus*** **Nrf2.**
(TIF)

Figure S5 **AD1 is dynamically repartitioned out of membranes into the cyto/nucleoplasmic side.**
(TIF)

Figure S6 **The CRAC4/TMp-adjoining sequences contribute to positive and negative regulation of Nrf1, with its net positive regions being primarily retained in the cyto/nucleoplasmic sides of membranes.**
(TIF)

Box S1 **Definition of the major domains and motifs abbreviated in this study.**
(DOC)

## References

[1] von Heijne G (2006) Membrane-protein topology. Nat. Rev. Mol. Cell Biol 7: : 909–918. PMID:17139331 10.1038/nrm2063 17139331

[2] Ye J, Rawson RB, Komuro R, Chen X, Dave UP, et al. (2000) ER stress induces cleavage of membrane-bound ATF6 by the same proteases that process SREBPs. Mol. Cell 6: : 1355–1364. PMID:11163209 10.1016/s1097-2765(00)00133-7 11163209

[3] Brown MS, Ye J, Rawson RB, Goldstein JL (2000) Regulated intramembrane proteolysis: a control mechanism conserved from bacteria to humans. . Cell 100: : 391–398. PMID:10693756 10.1016/s0092-8674(00)80675-3 10693756

[4] Wolfe MS, Kopan R (2004) Intramembrane proteolysis: theme and variations. Science 305: : 1119–1123. PMID:15326347 10.1126/science.1096187 15326347

[5] Zhang Y, Kobayashi A, Yamamoto M, Hayes JD (2009) The Nrf3 transcription factor is a membrane-bound glycoprotein targeted to the endoplasmic reticulum through its N-terminal homology box 1 sequence. J. Biol. Chem. 284: : 3195–3210. PMID:19047052 10.1074/jbc.M805337200 19047052

[6] Zhang Y, Lucocq JM, Hayes JD (2009) The Nrf1 CNC/bZIP protein is a nuclear envelope-bound transcription factor that is activated by t-butyl hydroquinone but not by endoplasmic reticulum stressors. Biochem. J. 418: : 293–310. PMID:18990090 10.1042/BJ20081575 18990090

[7] Zhang Y, Lucocq JM, Yamamoto M, Hayes JD (2007) The NHB1 (N-terminal homology box 1) sequence in transcription factor Nrf1 is required to anchor it to the endoplasmic reticulum and also to enable its asparagine-glycosylation. Biochem. J. 408: : 161–172. PMID:17705787 10.1042/BJ20070761 PMC2267355 17705787

[8] Sykiotis GP, Bohmann D (2010) Stress-activated cap‘n’collar transcription factors in aging and human disease. Sci. Signal. 3: : re3. PMID:20215646 10.1126/scisignal.3112re3 PMC2991085 20215646

[9] Steffen J, Seeger M, Koch A, Kruger E (2010) Proteasomal degradation is transcriptionally controlled by TCF11 via an ERAD-dependent feedback loop. Mol. Cell 40: : 147–158.PMID:20932482 10.1016/j.molcel.2010.09.012 20932482

[10] Radhakrishnan SK, Lee CS, Young P, Beskow A, Chan JY, et al. (2010) Transcription factor Nrf1 mediates the proteasome recovery pathway after proteasome inhibition in mammalian cells. Mol. Cell 38: : 17–28. PMID:20385086 10.1016/j.molcel.2010.02.029 PMC2874685 20385086

[11] Grimberg KB, Beskow A, Lundin D, Davis MM, Young P (2011) Basic leucine zipper protein Cnc-C is a substrate and transcriptional regulator of the Drosophila 26S proteasome. Mol. Cell Biol. 31: : 897–909. PMID:21149573 10.1128/MCB.00799-10 PMC3028647 21149573

[12] Li X, Matilainen O, Jin C, Glover-Cutter KM, Holmberg CI, et al. (2011) Specific SKN-1/Nrf stress responses to perturbations in translation elongation and proteasome activity. PLoS Genet. 7: : e1002119. PMID:21695230 10.1371/journal.pgen.1002119 PMC3111486 21695230

[13] Rushmore TH, Morton MR, Pickett CB (1991) The antioxidant responsive element. Activation by oxidative stress and identification of the DNA consensus sequence required for functional activity. J. Biol. Chem. 266: : 11632–11639. PMID:1646813 1646813

[14] Bean TL, Ney PA (1997) Multiple regions of p45 NF-E2 are required for b-globin gene expression in erythroid cells. Nucleic Acids Res. 25: : 2509–2515. PMID:9171106 10.1093/nar/25.12.2509 PMC146763 9171106

[15] Johnsen O, Murphy P, Prydz H, Kolsto AB (1998) Interaction of the CNC-bZIP factor TCF11/LCR-F1/Nrf1 with MafG: binding-site selection and regulation of transcription. Nucleic Acids Res. 26: : 512–520. PMID:9421508 10.1093/nar/26.2.512 PMC147270 9421508

[16] Farmer SC, Sun CW, Winnier GE, Hogan BL, Townes TM (1997) The bZIP transcription factor LCR-F1 is essential for mesoderm formation in mouse development. Genes Dev. 11: : 786–798. PMID:9087432 10.1101/gad.11.6.786 9087432

[17] Chan JY, Kwong M, Lu R, Chang J, Wang B, et al. (1998) Targeted disruption of the ubiquitous CNC-bZIP transcription factor, Nrf-1, results in anemia and embryonic lethality in mice. EMBO J. 17: : 1779–1787. PMID:9501099 10.1093/emboj/17.6.1779 PMC1170525 9501099

[18] Chen L, Kwong M, Lu R, Ginzinger D, Lee C, et al. (2003) Nrf1 is critical for redox balance and survival of liver cells during development. Mol. Cell Biol. 23: : 4673–4686. PMID:12808106 10.1128/MCB.23.13.4673-4686.2003 PMC164851 12808106

[19] Xu Z, Chen L, Leung L, Yen TS, Lee C, et al. (2005) Liver-specific inactivation of the Nrf1 gene in adult mouse leads to nonalcoholic steatohepatitis and hepatic neoplasia. Proc. Natl. Acad. Sci. USA 102: : 4120–4125. PMID:15738389 10.1073/pnas.0500660102 PMC554825 15738389

[20] Ohtsuji M, Katsuoka F, Kobayashi A, Aburatani H, Hayes JD, et al. (2008) Nrf1 and Nrf2 play distinct roles in activation of antioxidant response element-dependent genes. J. Biol. Chem. 283: : 33554–33562. PMID:18826952 10.1074/jbc.M804597200 PMC2662273 18826952

[21] Kobayashi A, Tsukide T, Miyasaka T, Morita T, Mizoroki T, et al. (2011) Central nervous system-specific deletion of transcription factor Nrf1 causes progressive motor neuronal dysfunction. Genes Cells 16: : 692–703. PMID:21554501 10.1111/j.1365-2443.2011.01522.x 21554501

[22] Lee CS, Lee C, Hu T, Nguyen JM, Zhang J, et al. (2011) Loss of nuclear factor E2-related factor 1 in the brain leads to dysregulation of proteasome gene expression and neurodegeneration. Proc. Natl. Acad. Sci. USA 108: : 8408–8413. PMID:21536885 10.1073/pnas.1019209108 PMC3100960 21536885

[23] Kim J, Xing W, Wergedal J, Chan JY, Mohan S (2010) Targeted disruption of nuclear factor erythroid-derived 2-like 1 in osteoblasts reduces bone size and bone formation in mice. Physiol. Genomics 40: : 100–110. PMID:19887580 10.1152/physiolgenomics.00105.2009 19887580

[24] Higgins LG, Kelleher MO, Eggleston IM, Itoh K, Yamamoto M, et al. (2009) Transcription factor Nrf2 mediates an adaptive response to sulforaphane that protects fibroblasts in vitro against the cytotoxic effects of electrophiles, peroxides and redox-cycling agents. Toxicol. Appl. Pharmacol. 237: : 267–280. PMID:19303893 10.1016/j.taap.2009.03.005 19303893

[25] Xiao H, Lü F, Stewart D, Zhang Y (2013) Mechanisms underlying chemopreventive effects of flavonoids via multiple signaling nodes within Nrf2-ARE and AhR-XRE gene regulatory networks. Curr. Chem. Biol. 7: , 151–176.

[26] Chan K, Lu R, Chang JC, Kan YW (1996) NRF2, a member of the NFE2 family of transcription factors, is not essential for murine erythropoiesis, growth, and development. Proc. Natl. Acad. Sci. USA 93: : 13943–13948. PMID:8943040 10.1073/pnas.93.24.13943 PMC19474 8943040

[27] Kim HM, Do CH, Lee DH (2010) Taurine reduces ER stress in C. elegans. J. Biomed. Sci. 17 Suppl 1: S26. PMID:20804601 10.1186/1423-0127-17-S1-S26 PMC2994371 20804601

[28] Zhang Y, Crouch DH, Yamamoto M, Hayes JD (2006) Negative regulation of the Nrf1 transcription factor by its N-terminal domain is independent of Keap1: Nrf1, but not Nrf2, is targeted to the endoplasmic reticulum. Biochem. J. 399: : 373–385. PMID:16872277 10.1042/BJ20060725 PMC1615900 16872277

[29] Zhang Y, Hayes JD (2013) The membrane-topogenic vectorial behaviour of Nrf1 controls its post-translational modification and transactivation activity. Sci. Rep. 3;2006: 1–16 DOI: 10.1038/srep02006. PMID:23774320 10.1038/srep02006 PMC3684815 23774320

[30] Tsuchiya Y, Morita T, Kim M, Iemura S, Natsume T, et al. (2011) Dual Regulation of the Transcriptional Activity of Nrf1 by b-TrCP- and Hrd1-Dependent Degradation Mechanisms. Mol. Cell Biol. 31: : 4500–4512. PMID:21911472 10.1128/MCB.05663-11 PMC3209242 21911472

[31] Witte MD, Horst D, Wiertz EJ, van der Marel GA, Overkleeft HS (2009) Synthesis and biological evaluation of a chitobiose-based peptide N-glycanase inhibitor library. J. Org. Chem. 74: : 605–616. PMID:19072094 10.1021/jo801906s 19072094

[32] Zhang Y, Cho YY, Petersen BL, Bode AM, Zhu F, et al. (2003) Ataxia telangiectasia mutated proteins, MAPKs, and RSK2 are involved in the phosphorylation of STAT3. J. Biol. Chem. 278: : 12650–12659. PMID:12562765 10.1074/jbc.M210368200 12562765

[33] Wang XJ, Hayes JD, Wolf CR (2006) Generation of a stable antioxidant response element-driven reporter gene cell line and its use to show redox-dependent activation of nrf2 by cancer chemotherapeutic agents. Cancer Res. 66: : 10983–10994. PMID:17108137 10.1158/0008-5472.CAN-06-2298 17108137

[34] Zhang Y, Hayes JD (2010) Identification of topological determinants in the N-terminal domain of transcription factor Nrf1 that control its orientation in the endoplasmic reticulum membrane. Biochem. J. 430: : 497–510. PMID:20629635 10.1042/BJ20100471 20629635

[35] Bailey D, Barreca C, O'Hare P (2007) Trafficking of the bZIP transmembrane transcription factor CREB-H into alternate pathways of ERAD and stress-regulated intramembrane proteolysis. Traffic 8: : 1796–1814. PMID:17875199 10.1111/j.1600-0854.2007.00654.x 17875199

[36] Afshar N, Black BE, Paschal BM (2005) Retrotranslocation of the chaperone calreticulin from the endoplasmic reticulum lumen to the cytosol. Mol. Cell Biol. 25: : 8844–8853. PMID:16199864 10.1128/MCB.25.20.8844-8853.2005 PMC1265792 16199864

[37] Kang SW, Rane NS, Kim SJ, Garrison JL, Taunton J, et al. (2006) Substrate-specific translocational attenuation during ER stress defines a pre-emptive quality control pathway. Cell 127: : 999–1013. PMID:17129784 10.1016/j.cell.2006.10.032 PMC3656606 17129784

[38] Gafvelin G, von Heijne G (1994) Topological “frustration” in multispanning E. coli inner membrane proteins. Cell 77: : 401–412. PMID:8181060 10.1016/0092-8674(94)90155-4 8181060

[39] Lorenz H, Hailey DW, Wunder C, Lippincott-Schwartz J (2006) The fluorescence protease protection (FPP) assay to determine protein localization and membrane topology. Nat. Protoc. 1: : 276–279. PMID:17406244 10.1038/nprot.2006.42 17406244

[40] Zhong Y, Fang S (2012) Live cell imaging of protein dislocation from the endoplasmic reticulum. J. Biol. Chem. 287: , 28057–28066. PMID:22722934 10.1074/jbc.M112.381798 PMC3431711 22722934

[41] van Geest M, Lolkema JS (2000) Membrane topology and insertion of membrane proteins: search for topogenic signals. Microbiol. Mol. Biol. Rev. 64: : 13–33. PMID:10704472 10.1128/mmbr.64.1.13-33.2000 PMC98984 10704472

[42] Zhang Y, Mattjus P, Schmid PC, Dong Z, Zhong S, et al. (2001) Involvement of the acid sphingomyelinase pathway in uva-induced apoptosis. J. Biol. Chem. 276: : 11775–11782. PMID:11278294 10.1074/jbc.M006000200 PMC2621016 11278294

[43] Yan Q, Lennarz WJ (1999) Oligosaccharyltransferase: a complex multisubunit enzyme of the endoplasmic reticulum. Biochem. Biophys. Res. Commun. 266: : 684–689. PMID:10603306 10.1006/bbrc.1999.1886 10603306

[44] Shibatani T, David LL, McCormack AL, Frueh K, Skach WR (2005) Proteomic analysis of mammalian oligosaccharyltransferase reveals multiple subcomplexes that contain Sec61, TRAP, and two potential new subunits. Biochemistry 44: : 5982–5992. PMID:15835887 10.1021/bi047328f 15835887

[45] Das MK, Sharma RS, Mishra V (2012) Induction of apoptosis by ribosome inactivating proteins: importance of N-glycosidase activity. Appl. Biochem. Biotechnol 166: : 1552–1561. PMID:22262020 10.1007/s12010-012-9550-x 22262020

[46] Piskacek S, Gregor M, Nemethova M, Grabner M, Kovarik P (2007) Nine-amino-acid transactivation domain: establishment and prediction utilities. Genomics 89: : 756–768. PMID:17467953 10.1016/j.ygeno.2007.02.003 17467953

[47] Sandholzer J, Hoeth M, Piskacek M, Mayer H, de Martin R (2007) A novel 9-amino-acid transactivation domain in the C-terminal part of Sox18. Biochem. Biophys. Res. Commun. 360: 370–374. PMID: 17603017 10.1016/j.bbrc.2007.06.095 17603017

[48] Stargell LA, Struhl K (1995) The TBP-TFIIA interaction in the response to acidic activators in vivo. Science 269: : 75–78. PMID:7604282 10.1126/science.7604282 7604282

[49] Zhang J, Hosoya T, Maruyama A, Nishikawa K, Maher JM, et al. (2007) Nrf2 Neh5 domain is differentially utilized in the transactivation of cytoprotective genes. Biochem. J. 404: : 459–466. PMID:17313370 10.1042/BJ20061611 PMC1896277 17313370

[50] Misaghi S, Pacold ME, Blom D, Ploegh HL, Korbel GA (2004) Using a small molecule inhibitor of peptide: N-glycanase to probe its role in glycoprotein turnover. Chem. Biol. 11: : 1677–1687. PMID:15610852 10.1016/j.chembiol.2004.11.010 15610852

[51] Hara H, Friedlander RM, Gagliardini V, Ayata C, Fink K, et al. (1997) Inhibition of interleukin 1beta converting enzyme family proteases reduces ischemic and excitotoxic neuronal damage. Proc. Natl. Acad. Sci. USA 94: : 2007–2012. PMID: 9050895 10.1073/pnas.94.5.2007 PMC20033 9050895

[52] Van Noorden CJ (2001) The history of Z-VAD-FMK, a tool for understanding the significance of caspase inhibition. Acta Histochem. 103: : 241–251. PMID:11482370 10.1078/0065-1281-00601 11482370

[53] O'Neill RA (1996) Enzymatic release of oligosaccharides from glycoproteins for chromatographic and electrophoretic analysis. J. Chromatogr. A 720: : 201–215. PMID:8601190 10.1016/0021-9673(95)00502-1 8601190

[54] Roth Z, Yehezkel G, Khalaila I (2012) Identification and quantification of protein glycosylation. Intl. J. Carbohydr. Chem. 2012: 1–10.

[55] Epand RM (2006) Cholesterol and the interaction of proteins with membrane domains. Prog. Lipid Res. 45: : 279–294. PMID:16574236 10.1016/j.plipres.2006.02.001 16574236

[56] Goder V, Bieri C, Spiess M (1999) Glycosylation can influence topogenesis of membrane proteins and reveals dynamic reorientation of nascent polypeptides within the translocon. J. Cell. Biol. 147: : 257–266. PMID:10525533 10.1083/jcb.147.2.257 PMC2174215 10525533

[57] Spiro RG (2004) Role of N-linked polymannose oligosaccharides in targeting glycoproteins for endoplasmic reticulum-associated degradation. Cell Mol. Life Sci. 61: : 1025–1041. PMID:15112051 10.1007/s00018-004-4037-8 PMC11138603 15112051

[58] Dowhan W, Bogdanov M (2009) Lipid-dependent membrane protein topogenesis. Annu. Rev. Biochem. 78: : 515–540. PMID:19489728 10.1146/annurev.biochem.77.060806.091251 PMC3033430 19489728

[59] Langosch D, Arkin IT (2009) Interaction and conformational dynamics of membrane-spanning protein helices. Protein Sci. 18: : 1343–1358. PMID:19530249 10.1002/pro.154 PMC2775205 19530249

[60] Skach WR (2009) Cellular mechanisms of membrane protein folding. Nat. Struct. Mol. Biol. 16: : 606–612. PMID:19491932 10.1038/nsmb.1600 PMC2814870 19491932

[61] Tsai B, Ye Y, Rapoport TA (2002) Retro-translocation of proteins from the endoplasmic reticulum into the cytosol. Nat. Rev. Mol. Cell Biol. 3: : 246–255PMID:11994744 10.1038/nrm780 11994744

[62] Vembar SS, Brodsky JL (2008) One step at a time: endoplasmic reticulum-associated degradation. Nat. Rev. Mol. Cell Biol. 9: : 944–957. PMID:19002207 10.1038/nrm2546 PMC2654601 19002207

[63] Chepelev NL, Bennitz JD, Huang T, McBride S, Willmore WG (2011) The Nrf1 CNC-bZIP protein is regulated by the proteasome and activated by hypoxia. PLoS One 6: : e29167. PMID:22216197 10.1371/journal.pone.0029167 PMC3244438 22216197

[64] Biswas M, Phan D, Watanabe M, Chan JY (2011) The Fbw7 tumor suppressor regulates nuclear factor E2 related factor 1 (Nrf1) transcription factor turnover through proteasome-mediated proteolysis. J. Biol. Chem 286: : 39282–39289. PMID:21953459 10.1074/jbc.M111.253807 PMC3234752 21953459

[65] Katiyar S, Joshi S, Lennarz WJ (2005) The retratranslocation protein Derlin-1 binds peptide:N-glycanase to the endoplasmic reticulum. Mol. Biol. Cell 16: : 4584–4594. PMID:16055502 10.1091/mbc.E05-04-0345 PMC1237066 16055502

[66] Allen MD, Buchberger A, Bycroft M (2006) The PUB domain functions as a p97 binding module in human peptide:N-glycanase. J. Biol. Chem. 281: : 25502–25508. PMID:16807242 10.1074/jbc.M601173200 16807242

[67] Suzuki T, Park MA, Kwofie MA, Lennarz WJ (2001) Rad23 provides a link between the Png1 deglycosylating enzyme and the 26S proteasome in yeast. J. Biol. Chem. 276: : 21601–21607. PMID:11259433 10.1074/jbc.M100826200 11259433

[68] Yoshida Y, Tanaka K (2010) Lectin-like ERAD player in ER and cytosol. Biochim. Biophys. Acta 1800: : 172–180. PMID:19665047 10.1016/j.bbagen.2009.07.029 19665047

[69] Swanson R, Locher M, Hochstrasser M (2001) A conserved ubiquitin ligase of the nuclear envelope/endoplasmic reticulum that functions in both ER-associated and Mata2 repressor degradation. Gene Dev. 15: : 2660–2674PMID:11641273 10.1101/gad.933301 PMC312819 11641273

[70] Bowie JU (2006) Flip-flopping membrane proteins. Nat. Struct. Mol. Biol. 13: : 94–96. PMID:16462808 10.1038/nsmb0206-94 16462808

[71] Holthuis JC, Levine TP (2005) Lipid traffic: floppy drives and a superhighway. Nat. Rev. Mol. Cell Biol. 6: : 209–220. PMID:15738987 10.1038/nrm1591 15738987

[72] Caterina JJ, Donze D, Sun CW, Ciavatta DJ, Townes TM (1994) Cloning and functional characterization of LCR-F1: a bZIP transcription factor that activates erythroid-specific, human globin gene expression. Nucleic Acids Res. 22: : 2383–2391PMID:8036168 10.1093/nar/22.12.2383 PMC523699 8036168

[73] Novotny V, Prieschl EE, Csonga R, Fabjani G, Baumruker T (1998) Nrf1 in a complex with fosB, c-jun, junD and ATF2 forms the AP1 component at the TNF alpha promoter in stimulated mast cells. Nucleic Acids Res. 26: : 5480–5485. PMID:9826775 10.1093/nar/26.23.5480 PMC147998 9826775

[74] Prieschl EE, Novotny V, Csonga R, Jaksche D, Elbe-Burger A, et al. (1998) A novel splice variant of the transcription factor Nrf1 interacts with the TNFalpha promoter and stimulates transcription. Nucleic Acids Res. 26: : 2291–2297. PMID:9580677 10.1093/nar/26.10.2291 PMC147553 9580677

[75] Katoh Y, Itoh K, Yoshida E, Miyagishi M, Fukamizu A, et al. (2001) Two domains of Nrf2 cooperatively bind CBP, a CREB binding protein, and synergistically activate transcription. Genes Cells 6: : 857–868. PMID:11683914 10.1046/j.1365-2443.2001.00469.x 11683914

[76] Blackwell TK, Bowerman B, Priess JR, Weintraub H (1994) Formation of a monomeric DNA binding domain by Skn-1 bZIP and homeodomain elements. Science 266: : 621–628. PMID:7939715 10.1126/science.7939715 7939715

