# Supplementary figures and images for: Transcription Factor Nrf1 Is Topologically Repartitioned across Membranes to Enable Target Gene Transactivation through Its Acidic Glucose-Responsive Domains
Source: PLoS One. 2014 Apr 2;9(4):e93458. doi: 10.1371/journal.pone.0093458 (PMC3973704; doi:10.1371/journal.pone.0093458)

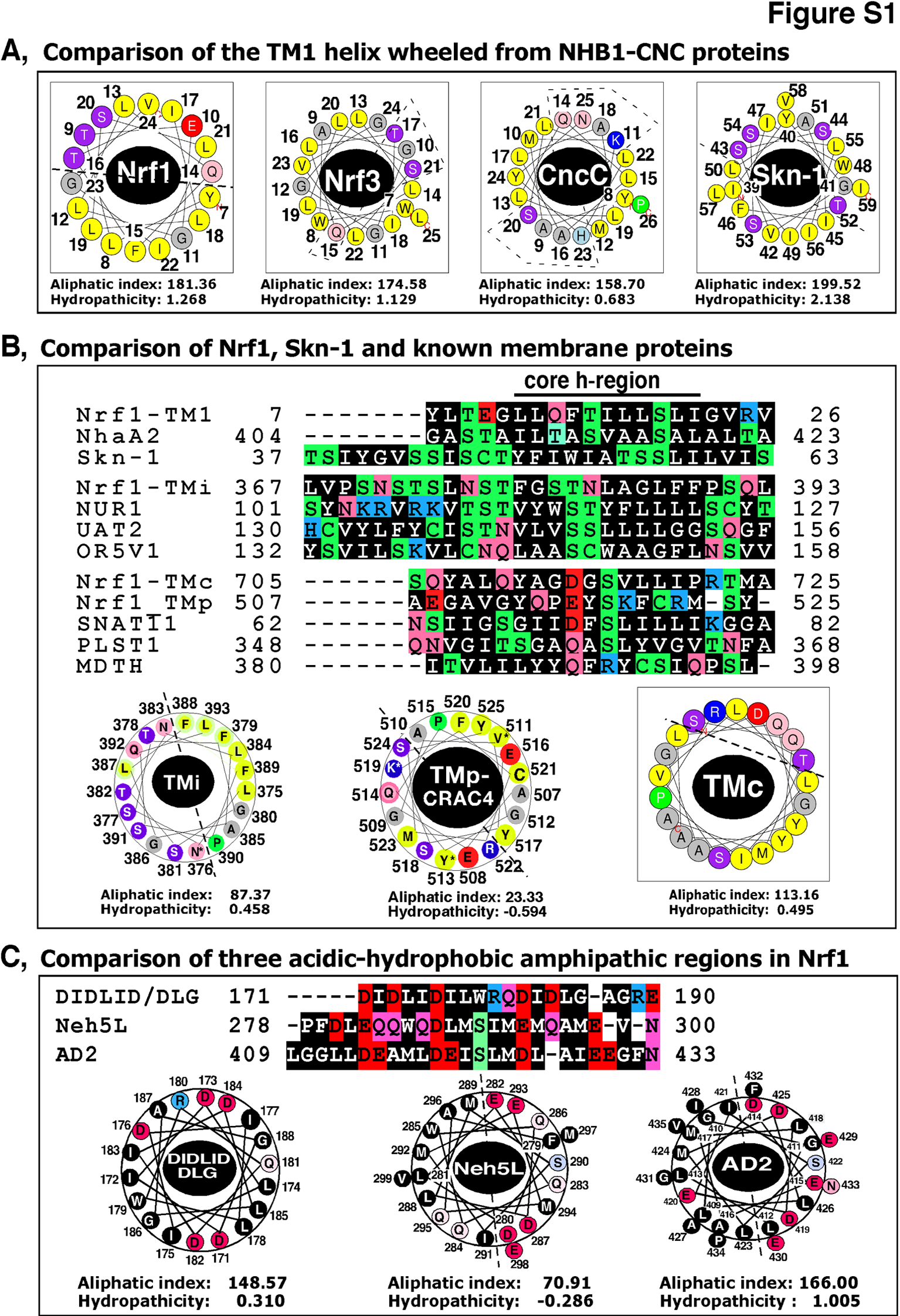

Supplement: Figure S1 — Comparison of topological determinants of NHB1-CNC factors within membranes. (TIF) [file pone.0093458.s001.tif]

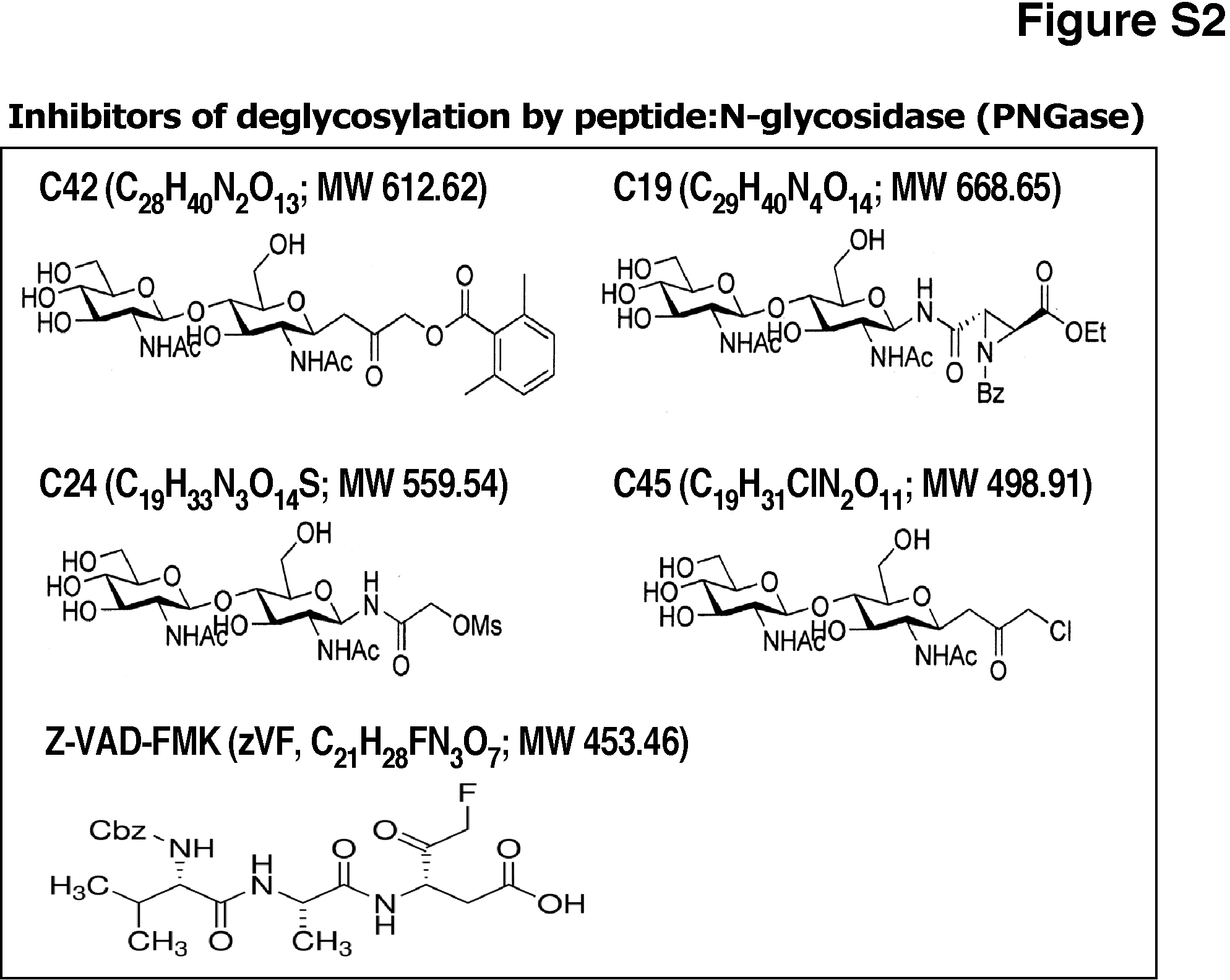

Supplement: Figure S2 — Structural differences between the chitobiose-based PNGase inhibitors and Z-VAD-FMK. (TIF) [file pone.0093458.s002.tif]

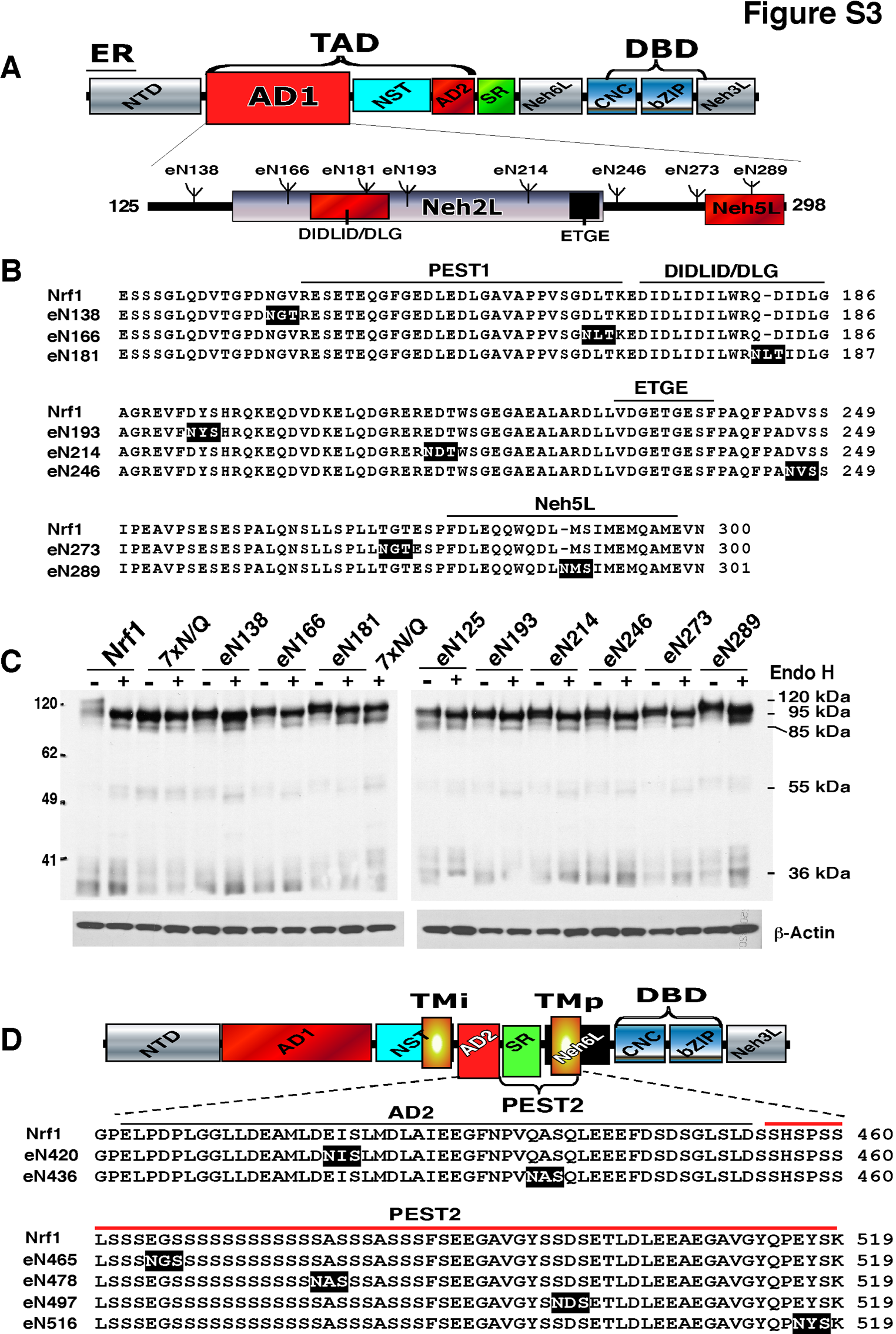

Supplement: Figure S3 — Engineered glycosylation mapping of AD1, AD2, SR and PEST2 within Nrf1. (TIF) [file pone.0093458.s003.tif]

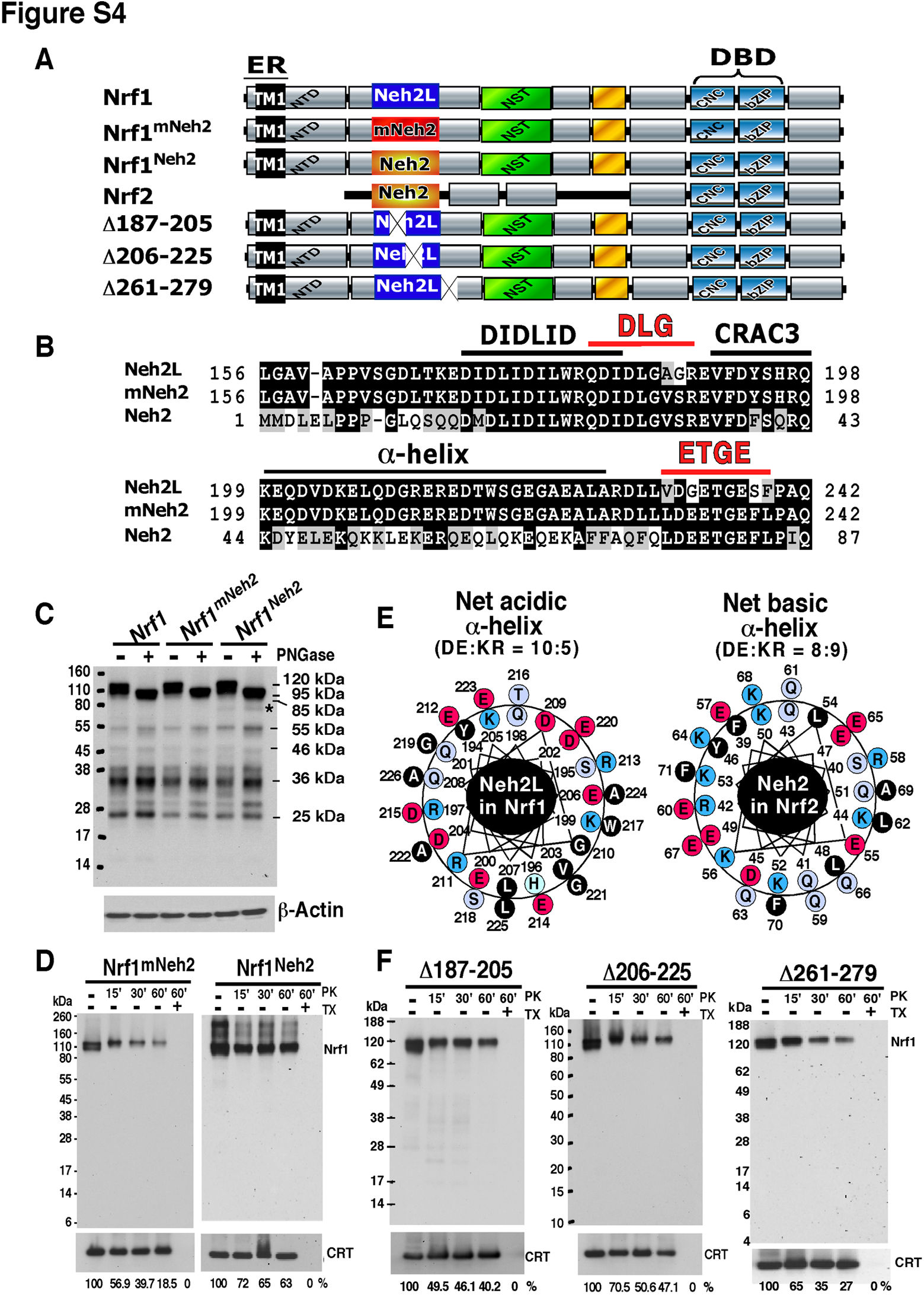

Supplement: Figure S4 — Opposing roles for Neh2L and Neh2 in regulating the function of Nrf1 versus Nrf2. (TIF) [file pone.0093458.s004.tif]

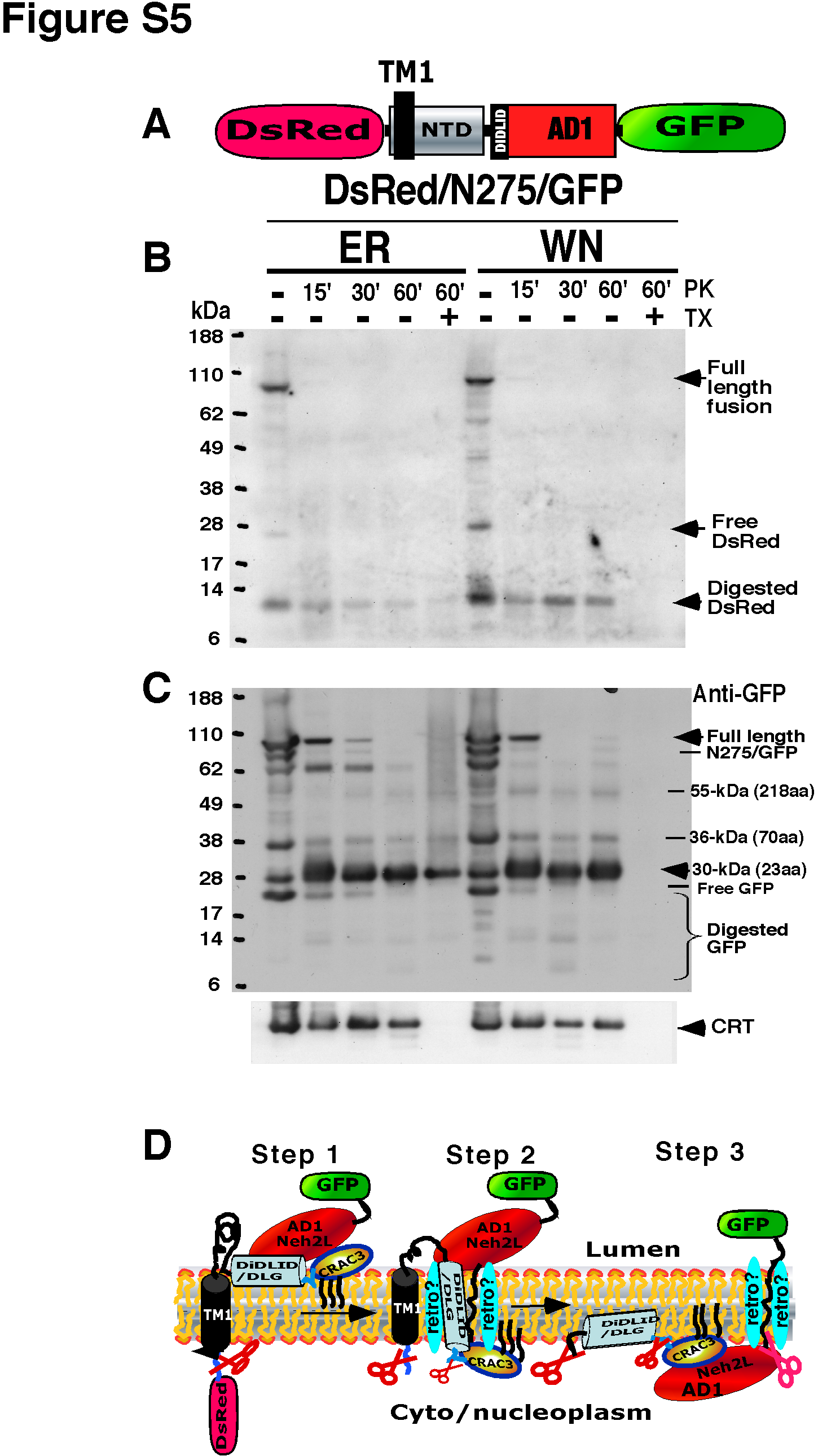

Supplement: Figure S5 — AD1 is dynamically repartitioned out of membranes into the cyto/nucleoplasmic side. (TIF) [file pone.0093458.s005.tif]

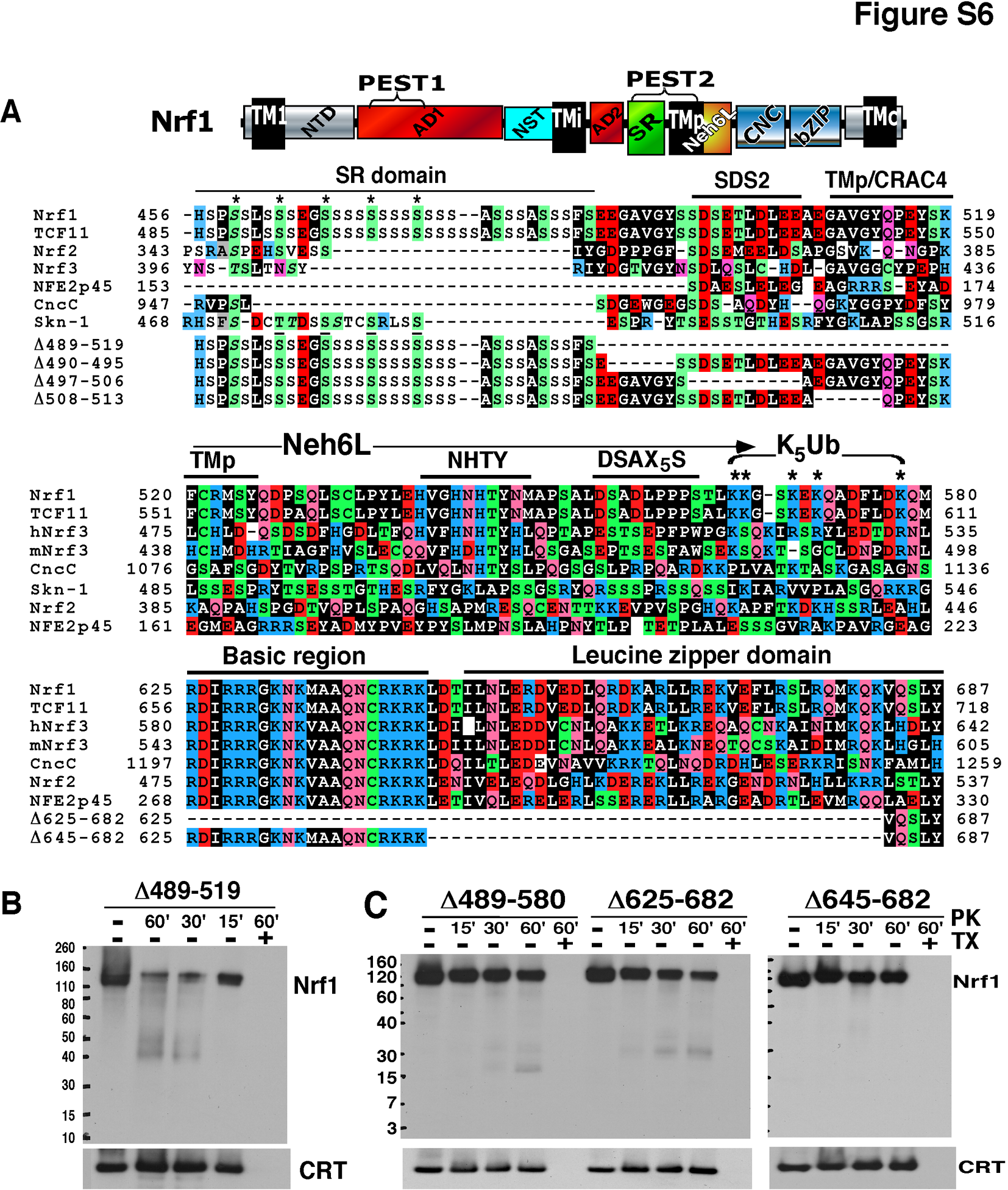

Supplement: Figure S6 — The CRAC4/TMp-adjoining sequences contribute to positive and negative regulation of Nrf1, with its net positive regions being primarily retained in the cyto/nucleoplasmic sides of membranes. (TIF) [file pone.0093458.s006.tif]
