# Supplementary material for: Transcription Factor Nrf1 Is Topologically Repartitioned across Membranes to Enable Target Gene Transactivation through Its Acidic Glucose-Responsive Domains
Source: PLoS One. 2014 Apr 2;9(4):e93458. doi: 10.1371/journal.pone.0093458 (PMC3973704; doi:10.1371/journal.pone.0093458)
Supplement: Box S1 — Definition of the major domains and motifs abbreviated in this study. (DOC) [file pone.0093458.s007.doc]

**Transcription factor Nrf1 is topologically repartitioned across membranes to enable target gene transactivation through its acidic glucose-responsive domains**

**Yiguo Zhang^1,2^,*****, Yonggang Ren^1^, Shaojun Li^1^, & John D. Hayes^2^**

^1^The NSFC-funded Laboratory of Cell Biochemistry and Gene Regulation, College of Medical Bioengineering and Faculty of Life Sciences, Chongqing University, Chongqing 400044, China; and ^2^Division of Cancer Research, Medical Research Institute, Ninewells Hospital & Medical School, University of Dundee, DD1 9SY, Scotland, UK

*Correspondence should be addressed to Yiguo Zhang ([yiguozhang@cqu.edu.cn, or](mailto:yiguozhang@cqu.edu.cn,or) [y.z.zhang@dundee.ac.uk](mailto:y.z.zhang@dundee.ac.uk))

**Box 1 Definition of the major domains and motifs abbreviated in this study**

**AD1** (acidic domain 1) functions as the major transactivation domain (TAD) in Nrf1, and comprises aa 125-298. This domain contains the PEST1 sequence (aa 141-170), the Neh2L subdomain (aa 156-242), the Cdc4 phospho-degron (CPD, ^267^LLSPLLT^273^) and the Neh5L subdomain (aa 280-298).

**AD2**  (acidic domain 2) contributes to transactivation activity of Nrf1, and is particularly important for the short Nrf1β/ LCR-F1 isoform. This domain includes an acidic-hydrophobic amphipathic region (aa 403-440) and a SDS1 (serine-aspartate-serine) motif (aa 441-455) that contains the ^447^DSGLS^451^ β-TrCP-binding degron.

**ARE** (antioxidant response element), also called the EpRE (electrophile response element), comprises the consensus sequence 5′-TGACnnnGC-3′ that is recognized by CNC-bZIP factors.

**CNC** (cap‘n’collar) domain was originally identified in a *Drosophila* transcription factor. The CNC family includes *C. elegans* Skn-1, the four vertebrate activators NF-E p45 subunit, Nrf1 (including its long form TCF11 and short form Nrf1b/LCR-F1), Nrf2 and Nrf3, and two distantly related repressors Bach1 and Bach2. This family shares a highly conserved 45-aa CNC domain (comprising aa 581-624 in Nrf1.

**CRAC** (cholesterol recognition amino acid consensus motif) adjoins membrane-associated segments to enable interaction of the protein with membrane lipids. CRAC1 (aa 62-70) and CRAC2 (aa 74-82) are located close to the TM1 region within the NTD of Nrf1. CRAC3 (^191^VxxYxxRxK^199^) lies immediately adjacent to the DIDLID/DLG element.

**Neh1L** (Nrf2-ECH homology 1-like) region contains both CNC and bZIP domains and functions as the DNA-binding domain (DBD).

**Neh2L** (Nrf2-ECH homology 2-like) subdomain is situated in the center of AD1 in Nrf1. It is overlapped N-terminally by the PEST1 sequence and is flanked C-terminally by the CPD and Neh5L regions. Importantly, the Neh2L contains DLG and ETGE motifs, but these do not target Nrf1 for the Keap1-mediated proteasomal degradation. The DLG motif overlaps with the DIDLID element; both are integrated together and therefore referred to as the DIDLID/DLG element (aa 171-186).

**Neh3L** (Nrf2-ECH homology 3-like) region, also called the C-terminal domain, includes a CRAC5 motif (aa 683-695) that lies adjacent to TMc (C-terminal transmembrane region, aa 705-725) and a putative ER retention signal (aa 730-741).

**Neh4L** (Nrf2-ECH homology 4-like) acts as a TAD in TCF11 but not in Nrf1. It is lost in Nrf1 by alternative splicing.

**Neh5L** (Nrf2-ECH homology 5-like) subdomain functions as an essential TAD. It shares homology with the DIDLID/ DLG element and an amphipathic region of AD2 region (aa 409-428).

**Neh6L** (Nrf2-ECH homology 6-like) domain is situated between SR (serine-repeats) domain and the DBD, and contributes to the negative regulation of Nrf1. The N-terminal 30-aa region of Neh6L that overlaps with the PEST2 sequence, contains SDS2 (serine-aspartate-serine motif 2), aa 497-506) and is adjacent to CRAC4/TMp (a proline-kinked hinge structure folded by aa 507-525).

**N/D** (asparagine-to-aspartate) mutagenesis was used to scan the NST domain for glycosylation and deglycosylation sites.

**N/Q** (asparagine-to-glutamine) mutagenesis was used to scan the NST domain for glycosylation sites. Glycosylation mapping was performed by introduction of engineered asparagine (eN) glycosylation consensus sites into other domains.

**NHB1** (N-terminal homology box 1) comprises aa 11-30 in the N-terminal domain (NTD, aa 1-124) of Nrf1. The NHB1 sequence is highly conserved with equivalents in TCF11, Nrf3, CncC and Skn-1. We therefore propose that they are grouped together as to the ‘NHB1-CNC’ subfamily of membrane-binding transcription factors.

**NST** (asparagine, serine and threonine-rich aa 299-400) domain is situated between AD1 and AD2. It exists as a glycodomain in the ER, and has a capability to function as a *bona fide* TAD, which would be exerted only after it is repartitioned out of membranes into the cyto/nucleoplasm.

**PEST** (proline, glutamic acid, serine and threonine-rich) sequence acts as a degron that targets the protein for calpain- and/or proteasome-mediated proteolysis. Besides the PEST1 sequence (aa 141-170) in the N-terminal one-third of AD1, the PEST2 sequence (aa 456-519) covers the entire SR domain comprising aa 454-488, the **SDS2** motif (aa 497-506), and the CRAC4/TMp core region (aa 508-519).

**SDS1**  (serine-aspartate-serine motif 1) region includes the ^447^DSGLS^451^ degron within the C-terminal side of AD2.

**Supplemental RESults**

The supplemental results that had been obtained from both experimental methods and bioinformatics analysis are described in the following six figure legends.

**Figure S1. Comparison of topological determinants of NHB1-CNC factors within membranes**.

(**A**) Comparison of the TM1 helices within Nrf1, Nrf3, CncC and Skn-1. The α-helices of NHB1-CNC factors were wheeled using the HeliQuest programme. Some of the polar and charged amino acids in the membrane-spanning helices enable an intramolecular interaction between TM1 and other semihydrophobic and/or amphipathic α-helices folded by other regions within the NHB1-CNC factor (below), as has been described for other transmembrane proteins. Three physico-chemical parameters related with the helical folding (i.e. aliphaticity, hydropathicity and instability indexes) were calculated using the ProParam tool (http://web.expasy.org/ protparam/). These bioinformatics analyses predict that Skn-1 is an integral transmembrane glycoprotein with the proposed topology. (**B**) An alignment of the hydrophobic, semihydrophobic amphipathic sequences from Nrf1 with known membrane-associated domains***.*** The α-helices of TMi, TMp and TMc were wheeled as shown. **(C)** Prediction of three acidic-hydrophobic amphipathic secondary structures formed by amino acids within and around the DIDLID/DLG, Neh5L and AD2 regions. Amino acid alignment of AD2, DIDLID/DLG and Neh5L revealed that a portion of AD2 (aa 409-433) shares 75% and 40% similarity with Neh5L (aa 278-298) and DIDLID/DLG (aa 171-188), respectively. However, the aliphaticity and hydropathicity of the amphipathic portion of AD2 are much greater than the respective values of Neh5L, DIDLID/DLG and TMc, and are close to those of TM1. Together with the experimental evidence provided in this study, we propose that AD2 can be wheeled into a more stable amphipathic helix than can Neh5L and the DIDLID/DLG element. This bioinformatic observation adds weight to the hypothesis that together AD2 and the TMi peptide regulate folding of Nrf1 within the luminal interface of membranes and/or its repartitioning out of membranes. According to current membrane-topological folding theory, it is plausible that the acidic-hydrophobic amphipathic DIDLID/DLG element, Neh5L and AD2 regions would exist in a non-structural loop when they are unfolded in solution. However, differential interaction of these three regions with the bilayer of membrane lipids enables them to fold into acidic-hydrophobic amphipathic α-helices that can flexibly change in different local environments. Comparison of their aliphaticity and hydropathicity indicates that the relatively stable AD2 and the DIDLID/DLG element may dynamically lie flat on the luminal interface or span across the membrane through an unidentified mechanism. The Neh5L region may be more flexible than either the DIDLID/DLG element or AD2, and thus might determine topological repartitioning of Nrf1 across membranes in order to activate its target genes.

**Figure S2. Structural differences between the chitobiose-based PNGase inhibitors and Z-VAD-FMK.**

The chitobiose compounds 19, 24, and 45, abbreviated as C19, C24, and C45, were used to inhibit peptide:N-glycanase (PNGase). Their chemical properties have been reported previously. Compound 42 (C42) was used as a negative control. The caspase inhibitor Z-VAD-fmk was also identified as an inhibitor of PNGase.

**Figure S3. Engineered glycosylation mapping of AD1, AD2, SR and PEST2 within Nrf1.**

(**A**) Diagram illustrating the position of eight engineered glycosylation consensus asparagines (eN) within AD1**.** The *upper cartoon* shows nine structural domains of Nrf1. Its NTD contains the ER targeting signal. The AD1, NST and AD2 domains act as three distinct TADs. Distinct functional regions and motifs within AD1 are indicated in the *lower cartoon*. Importantly, eight engineered asparagine (eN) glycosylation consensus sites that were created within the AD1 of Nrf1^1-7N/Q^ are shown. (**B**) Amino acid alignment of AD1 in Nrf1^1-7N/Q^ with its eN mutants showing sequence changes. The engineered asparagine-linked glycosylation consensus sites have been placed on a black background. In addition, the horizontal bar above the amino acids shows the portions of the PEST1, DIDLID/DLG, ETGE and Neh5L regions in Nrf1. (**C**) Translocation of AD1 into the lumen of the ER. Each of expression constructs for Nrf1^1-7N/Q^ or its eN mutants was transfected overnight into COS-1 cells, which were then allowed to recover in fresh complete media for 24 h. The cell lysates (30 μg of protein) were subjected to *in vitro* deglycosylation reactions with Endo H (500 unit). Thereafter, the reactants were analyzed by LDS/NuPAGE containing 7% Tris-Acetate gel, followed by western blotting. The immunoblot shown represents an uncropped version of that presented in Figure 5A (*upper panel*). Their transactivation activity is presented in Figure 5B. (**D**) Design of glycosylation consensus asparagines engineered into AD2, SR and PEST2. Amino acid sequences of AD2 and the SR-containing PEST2 regions in Nrf1 are shown, aligned with those of its eN mutants that were introduced into Nrf1^1-7N/Q^. Asparagine-linked glycosylation consensus sites have been placed on a black background. These eN mutant proteins were characterized by measuring ARE-driven lucifearse activity (Figure 5D), as well as western blotting following delycosylation digestion with Endo H or PNGase F (Figure 5C).

**Figure S4. Opposing roles for Neh2L and Neh2 in regulating the function of Nrf1 *versus* Nrf2.**

**(A)** Schematic structures of Nrf1, Nrf1^mNeh2^, Nrf1^Neh2^, and other mutants, along with Nrf2. The Nrf1^mNeh2^ mutant contains a mimicked Neh2 (mNeh2) domain, insofar as the regions in Nrf1 equivalent to the two Keap1-binding DLG and ETGE motifs in Nrf2 were mutated to provide a sequence identical to that in Nrf2; it was created by several rounds of PCR-directed mutagenesis. The Nrf1^Neh2^ mutant was made by PCR-cloning of the Neh2 domain in Nrf2 to replace Neh2L in Nrf1. **(B)** Amino acid alignment of the Neh2L, Neh2, and mNeh2 sequences. Based on the structure of Neh2 deduced by nuclear magnetic resonance (NMR) , a homologous a-helical region between the two Keap1-binding sites DLG and ETGE is indicated within Neh2L and Neh2. Importantly, Neh2L contains a CRAC3 motif (^191^VxxYxxRxK^199^, in which the underlined Tyr residue can anchor an associated peptide into cholesterol-rich membranes ). However, a CRAC motif or an equivalent is not represented in the Neh2 domain of Nrf2. **(C)** No obvious differences were observed between the Nrf1, Nrf1^mNeh2^, and Nrf1^Neh2^ before and after deglycosylation**.**  The expression vector for Nrf1^Neh2^ yielded an extra unstable 80-kDa protein (indicated by*). Total lysates of cells that expressed ectopic Nrf1, Nrf1^mNeh2^ and Nrf1^Neh2^ were incubated with (+) or without (-) 500 units PNGase F for 60 min at 37°C. The reaction products were resolved by 4-12% LDS/NuPAGE and visualized by immunoblotting. **(D)** Repartitioning of Nrf1 is enhanced by mNeh2, but is impeded by Neh2 from Nrf2. The time-course of membrane protection reactions were carried out using the intact ER fraction puried from COS-1 cells expressing Nrf1^mNeh2^ or Nrf1^Neh2^, followed by western blotting with antibodies against Nrf1β as described for Figure 6B. The membrane protection assay revealed that Nrf1^Neh2^ exhibited behaviour similar to that of the Nrf1^Δ280-298^ mutant lacking Neh5L (herein and Figure 6C), but the dynamic repartitioning of Nrf1 within and around membranes appeared to be unaffected by the substitute Nrf1^mNeh2^ that contained the DLG and ETGE motifs of Nrf2. The difference in the membrane behaviour may be attributed to the presence of the putative CRAC3 sequence in Nrf1 rather than Nrf2. **(E)** Two similar yet distinct α−helices are folded by Neh2L and Neh2. The *left* wheel represents a net acidic α-helix that is predicted to be folded by amino acids 194-226 in the Neh2L of Nrf1. The *right* wheel shows a net basic α-helix folded by amino acids 39-71 of Neh2, based on the NMR evidence for the structure of Neh2 . Notably, the nonapeptide ^191^VFDYSHRQK^199^ (called CRAC3) is situated at the N-terminal border of the Neh2L α-helical region close to the DIDLID/DLG element in Nrf1. The putative cholesterol-binding motif CRAC3 may influence the folding and repartitioning of DIDLID/DLG and Neh2L. **(F)** Repartitioning of Nrf1 is partially modulated by aa 187-205 and aa 206-225, but not by aa 261-279. COS-1 cells were grown in a 100-mm dish and transfected for 18 h with 6 μg of an expression construct for Nrf1^Δ187-205^ (lacking CRAC3 and flanking hexapeptide in Neh2L), Nrf1^Δ206-225^ (lacking most of the Neh2L α-helical region) or Nrf1^Δ261-279^ (lacking CPD). Following recovery from transfection, the cells were then subjected to subcellular fractionation, followed immediately by membrane proteinase protection reactions that were carried out for 15, 30, or 60 min in 20 μl of 1× isotonic buffer containing 50 μg/ml (+) of PK in the presence (+) or absence (−) of 1% TX, together with 100 μg protein from the intact ER fractions. The reaction products (10 μg of protein diluted in 10 μl of 1× loading buffer) were resolved in a 4-12% LDS/NuPAGE Tris-Bis gel and Nrf1 protein identified by western blotting with antibodies against Nrf1β, as described previously. The antibody-blotted nitrocellulose membrane was stripped and re-probed with antibodies against calreticulin (CRT). The intensity of these western blots was calculated using the Quantity One® software. The relative amount of Nrf1 after PK digestion was estimated by dividing its immunoreactive band intensity with that obtained for CRT, and then the resulting values were normalized to the total non-digested amounts and shown as percentages (%) at the bottom. Some of the results obtained from membrane protection reactions are shown in the text (Figure 6C, n = 4).

**Figure S5. AD1 is dynamically repartitioned out of membranes into the cyto/nucleoplasmic side.**

**(A**) A structural diagram of DsRed/N275/GFP. The triple sandwich fusion protein was engineered by inserting nucleotide sequences encoding N275 (retaining the N-terminal 275 amino acids of Nrf1) between the cDNAs for DsRed2 and GFP within the pDsRed2-GFP vector through the SalI/KpnI multiple cloning sites . The N275 portion of Nrf1 covers the NTD and most of its AD1, though it lacks the Met-rich Neh5L region so as to eliminate the putative internal in-frame translation of free GFP-fused protein. The locations of TM1, and its flanking DsRed and GFP epitopes are indicated. In addition, the DIDLID/DLG element is contained within Neh2L of the AD1 portion. **(B,C**) Dynamic repartitioning of the DIDLID/DLG element-containing N275 mini-protein out of membranes. Subcellular fractionation was used to isolate the intact ER and whole nuclei (WN) from cells expressing the DsRed/N275/GFP sandwich fusion proteins. Subsequently, equal amounts (50 μg of protein in 40 μl of 1× isotonic buffer) of ER and WN were examined by membrane proteinase protection reactions that were carried out by incubating (on ice) each of the subcellular fractions with 50 μg/ml of PK in the presence (+) or absence (−) of 1% TX for 5, 15, 30 and 60 min. The reaction products were identified by western blotting with antibodies against DsRed (***B***) or GFP (***C***). The DsRed antibody-blotted nitrocellulose membrane was stripped and re-probed with antibodies against GFP and CRT. Four PK-digested peptides of 62, 55, 36 and 30 kDa were estimated to contain about 275, 218, 70 and 23 aa of Nrf1, respectively. It is therefore concluded that besides the cytoplasmic n-region of TM1, three other sites around residues 57, 205 and 252 are susceptible to PK digestion. This finding indicates that CRAC1/2 (aa 55-82) in NTD, CRAC3 (aa191-199) adjoining the DIDLID/DLG element, and the proline-rich hinge (PRH) region close to the ETGE motif in Neh2L may promote the dynamic repartitioning of N275 out of membranes. **(D**) A proposed model to explain the dynamic repartitioning of N275 in the sandwich fusion context. The membrane-topology of DsRed/N275/GFP is determined by its TM1 region and adopts an N_cyto_/C_lum_ orientation spanning membranes. Therefore, the DsRed epitope is retained on the cyto/nucleoplasmic side so that they are not protected by membranes against digestion by PK (scissors). By contrast, other regions (e.g. AD1) fused with GFP are transiently translocated into the ER lumen where they were to certain extents protected by membranes. However, a multiple PK-digested peptide ladder of N275-GFP indicates that the DIDLID/DLG-adjoining regions within Neh2L are dynamically repartitioned and/or retrotranslocated from the luminal side into the cyto/nucleoplasmic side where it is not protected by membranes from proteolysis. Thus cleavage of DsRed/N275/GFP can occur in three steps that are depicted in the cartoon. Based on published articles, together with bioinformatic analysis and the above experimental data, we predict that when the flexible DIDLID/DLG element interacts with membranes through the CRAC3 motif and/or with other semihydrophobic amphipathic helices within membranes, it might fold into an acidic-hydrophobic amphipathic helix (Figure S1C) that could lie flat on the interfaces of the membrane lipid bilayer or flip out of membrane lipids, in particular shingolipid and cholesterol enriched in the detergent-resistant membrane microdomain, as described elsewhere.

**Figure S6. The CRAC4/TMp-adjoining sequences contribute to positive and negative regulation of Nrf1, with**

**Its net positive regions being primarily retained in the cyto/nucleoplasmic sides of membranes.**

**(A)** The upper putative PEST2 sequence spans the SR and Neh6L domains. The PEST2 sequence contains the serine-repeat (SR) domain and the N-terminal one-third of Neh6L. The alignment of PEST2 amino acid sequences from different CNC transcription factors indicates that SR is specific for Nrf1 but is not present in the other homologous proteins. However, the SDS2-adjcent sequence is represented in all CNC factors, but the CRAC4 (^511^VxYxxxK^519^) motif appears to be specific for Nrf1. Conserved GSK-3 phophorylation sites are situated in the N-terminal border of the SR region. Like AD2, the SR region contributes to stimulation of Nrf1-mediated transactivation of target genes by glucose deprivation, whilst PEST2 and SDS2 contribute to the negative regulation of Nrf1. **(B)** The lower alignment of amino acids covering the net positive regions of Nrf1 reveals that the Neh6L domain is less conserved than the bZIP domain amongst the family members. In Nrf1, the net positive portion of Neh6L comprises NHTY (aa 540-548) and DSAX_5_S (aa 554-562), immediately followed by a lysine-rich cluster (K_5_-UB, aa 565-578, that has potential to be ubiquitylated by SCF^β-TrCP^). Both Cys^521^ and Cys^533^ are located within and around the TMp sequence, and possibly respond to redox stress. **(C)** The CRAC4-adjoining TMp sequence restricts dynamic repartitioning of Nrf1 out of membranes**.** Reactions to determine the time-course of membrane protection of Nrf1 were performed as described in Figure S8. In these experiments, the ER fraction of cells expressing the Nrf1^Δ489-519^ mutant (lacking the core TMp region ^507^AEGAVGYQPEYSK^519^ and its N-terminally flanking negative SDS2 peptide) was exposed to PK for various periods of time. The Nrf1^Δ489-519^ mutant was almost completely digested by PK within 30 min, indicating that it is dynamically repartitioned out of ER membranes into the cyto/nucleoplasm, allowing it to transactivate target gene expression (Figure 6F). **(D**) Lack of a net positive region allows Nrf1 mutant proteins to position in the lumen and to be retained therein. Intact ER fractions were purified from cells expressing Nrf1^Δ489-580^ (lacking the entire Neh6L), Nrf1^Δ625-682^ (lacking the entire bZIP), or Nrf1^Δ645-682^ (lacking the leucine zipper domain). Subsequently, equal amounts (50 μg of protein in 40 μl of 1× isotonic buffer) of ER were examined by membrane protection assays that were carried out by incubating (on ice) each of the subcellular fractions with 50 μg/ml of PK in the presence (+) or absence (−) of 1% TX for 15, 30 and 60 min. The reaction products were examined by immunoblotting with antibodies against Nrf1β (*upper*). The antibody-blotted nitrocellulose membrane was stripped and re-probed with CRT antibodies (*lower*). The intensity of blots was determined by the ImageJ software and calculated as shown graphically (Figure 6E).

**Supplemental References**

1. von Heijne G (2006) Membrane-protein topology. Nat Rev Mol Cell Biol 7: 909-918.

2. Langosch D, Arkin IT (2009) Interaction and conformational dynamics of membrane-spanning protein helices. Protein Sci 18: 1343-1358.

3. Dowhan W, Bogdanov M (2009) Lipid-dependent membrane protein topogenesis. Annu Rev Biochem 78: 515-540.

4. Bogdanov M, Xie J, Dowhan W (2009) Lipid-protein interactions drive membrane protein topogenesis in accordance with the positive inside rule. J Biol Chem 284: 9637-9641.

5. Bowie JU (2005) Solving the membrane protein folding problem. Nature 438: 581-589.

6. Witte MD, Horst D, Wiertz EJ, van der Marel GA, Overkleeft HS (2009) Synthesis and biological evaluation of a chitobiose-based peptide N-glycanase inhibitor library. J Org Chem 74: 605-616.

7. Misaghi S, Pacold ME, Blom D, Ploegh HL, Korbel GA (2004) Using a small molecule inhibitor of peptide: N-glycanase to probe its role in glycoprotein turnover. Chem Biol 11: 1677-1687.

8. Tong KI, Katoh Y, Kusunoki H, Itoh K, Tanaka T, et al. (2006) Keap1 recruits Neh2 through binding to ETGE and DLG motifs: characterization of the two-site molecular recognition model. Mol Cell Biol 26: 2887-2900.

9. Epand RM (2008) Proteins and cholesterol-rich domains. Biochim Biophys Acta 1778: 1576-1582.

10. Zhang Y, Hayes JD (2010) Identification of topological determinants in the N-terminal domain of transcription factor Nrf1 that control its orientation in the endoplasmic reticulum membrane. Biochem J 430: 497-510.

11. Zhang Y, Lucocq JM, Hayes JD (2009) The Nrf1 CNC/bZIP protein is a nuclear envelope-bound transcription factor that is activated by t-butyl hydroquinone but not by endoplasmic reticulum stressors. Biochem J 418: 293-310.

12. Holthuis JC, Levine TP (2005) Lipid traffic: floppy drives and a superhighway. Nat Rev Mol Cell Biol 6: 209-220.

13. Bowie JU (2006) Flip-flopping membrane proteins. Nat Struct Mol Biol 13: 94-96.
